# Pharmacometric and Electrocardiographic Evaluation of Chloroquine and Azithromycin in Healthy Volunteers

**DOI:** 10.1002/cpt.2665

**Published:** 2022-06-22

**Authors:** Palang Chotsiri, Joel Tarning, Richard M. Hoglund, James A. Watson, Nicholas J. White

**Affiliations:** ^1^ Mahidol‐Oxford Tropical Medicine Research Unit, Faculty of Tropical Medicine Mahidol University Bangkok Thailand; ^2^ Centre for Tropical Medicine and Global Health, Nuffield Department of Medicine Oxford University Oxford UK

## Abstract

Chloroquine and azithromycin were developed in combination for the preventive treatment of malaria in pregnancy, and more recently were proposed as coronavirus disease 2019 (COVID‐19) treatment options. Billions of doses of chloroquine have been administered worldwide over the past 70 years but concerns regarding cardiotoxicity, notably the risk of torsades de pointes (TdP), remain. This investigation aimed to characterize the pharmacokinetics and electrocardiographic effects of chloroquine and azithromycin observed in a large previously conducted healthy volunteer study. Healthy adult volunteers (*n* = 119) were randomized into 5 arms: placebo, chloroquine alone (600 mg base), or chloroquine with either 500 mg, 1,000 mg, or 1,500 mg of azithromycin all given daily for 3 days. Chloroquine and azithromycin levels, measured using liquid‐chromatography tandem mass spectrometry, and electrocardiograph intervals were recorded at frequent intervals. Time‐matched changes in the PR, QRS, and heart rate‐corrected JT, and QT intervals were calculated and the relationship with plasma concentrations was evaluated using linear and nonlinear mixed‐effects modeling. Chloroquine and azithromycin pharmacokinetics were described satisfactorily by two‐ and three‐compartment distribution models, respectively. No drug–drug interaction between chloroquine and azithromycin was observed. Chloroquine resulted in concentration‐dependent prolongation of the PR, QRS, JTc and QTc intervals with a minimal additional effect of azithromycin. QRS widening contributed ~ 28% of the observed QT prolongation. Chloroquine causes significant concentration‐dependent delays in both ventricular depolarization and repolarization. Co‐administration of azithromycin did not significantly increase these effects. The arrhythmogenic risk of TdP associated with chloroquine may have been substantially overestimated in studies which did not separate electrocardiograph QRS and JT prolongation.

**Study Highlights**

WHAT IS THE CURRENT KNOWLEDGE ON THE TOPIC?

Chloroquine is one of the most extensively used antimalarial drugs worldwide. Azithromycin is a very widely used antibiotic. They have very good safety records. The two drugs together have been evaluated in malaria prevention and coronavirus disease 2019 (COVID‐19) treatment. Both prolong the electrocardiograph QT interval. It has been widely hypothesized that this could result in cardiotoxicity (ventricular tachyarrhythmias).

WHAT QUESTION DID THIS STUDY ADDRESS?

This study examined whether chloroquine and azithromycin have any pharmacokinetic or cardiac pharmacodynamic interaction. It also characterized the concentration‐effect relationships for electrocardiographic effects to inform risk assessments.

WHAT DOES THIS STUDY ADD TO OUR KNOWLEDGE?

Chloroquine was found to cause concentration‐dependent prolongation of the PR, QRS, JTc, and QTc intervals without any significant additional effects of azithromycin co‐administration. The concentration‐dependent QRS widening (ventricular depolarization) contributed over one quarter of the QT prolongation.

HOW MIGHT THIS CHANGE CLINICAL PHARMACOLOGY OR TRANSLATIONAL SCIENCE?

Risks of iatrogenic torsades de pointes with chloroquine and hydroxychloroquine have been inferred from the extent of QTc prolongation. These have previously assumed that QT prolongation derives solely from prolongation of ventricular repolarization (JT prolongation), whereas over one quarter derives from QRS widening. The risks of ventricular arrhythmia resulting from therapeutic doses of chloroquine and hydroxychloroquine have been overestimated.

At the start of the coronavirus disease 2019 (COVID‐19) pandemic, there was extensive speculation, intense politicization, and a wide diversity of opinions over the potential value of the 4‐aminoquinolines, hydroxychloroquine and chloroquine, and the macrolide azithromycin, in the prevention and treatment of COVID‐19. This originated with a small study from France claiming therapeutic benefit for a combination of hydroxychloroquine and azithromycin.^1^, ^2^ Following this report, there was widespread use of these drugs in combination across the world, and many countries included them in treatment recommendations. However, opinion swung strongly against hydroxychloroquine following a high‐profile publication describing a large multinational investigation in which hydroxychloroquine treatment of patients with hospitalized with COVID‐19 was associated with lethal cardiotoxicity. This alarming study was soon shown to be fabricated.^3^ It was retracted swiftly but, together with the extreme politicization, it left a “toxic milieu” which compromised subsequent research and objective evaluation of the 4‐aminoquinolines.^4^ It is now clear from large randomized controlled trials, that neither hydroxychloroquine nor azithromycin benefit patients hospitalized with COVID‐19.^5^, ^6^ Whether these drugs provide benefit earlier in the course of disease, or in chemoprevention, remains to be determined.^4^

Before the COVID‐19 pandemic, chloroquine and hydroxychloroquine had been used extensively over the past 70 years for malaria and rheumatic diseases, respectively,^7^, ^8^ and azithromycin has been the first‐line treatment for bacterial infections for over 2 decades. These are among the most widely used anti‐infective drugs ever, and they have a very good record of tolerability and safety. Several billion chloroquine treatments have been given since the 1950s. Azithromycin has been given to hundreds of millions of healthy children in trachoma elimination programs.^9^ The main concern for both drugs has been the potential for cardiovascular toxicity and, in particular, the potential for lethal ventricular tachycardia (torsade de pointes (TdP)) as a consequence of delayed ventricular repolarization (reflected in the electrocardiogram as a prolonged QT interval).^10^, ^11^, ^12^, ^13^ Some observational studies suggested potentiation of QT prolongation when azithromycin and hydroxychloroquine are combined for the treatment of hospitalized patients with COVID‐19,^14^, ^15^ but others did not.^16^ Concern over the potential for cardiotoxicity in COVID‐19 prevention or treatment has resulted in numerous commentaries, reviews, expressions of concern, and advisory statements, but little detailed prospective investigation. Meanwhile, a series of large randomized controlled trials and observational studies have provided reassuring evidence on cardiovascular safety.^4^, ^5^, ^6^, ^17^, ^18^, ^19^

During the development of chloroquine and azithromycin as a potential combination for intermittent preventive treatment for malaria in pregnancy, a large and detailed evaluation was performed in healthy volunteers, which provided important pharmacometric data to characterize the effect of chloroquine on the electrocardiographic intervals and the interaction with azithromycin. These data were used to evaluate the population pharmacokinetic and electrocardiographic effects of chloroquine and azithromycin.

## METHODS

This was an internal pharmacometric study conducted by Pfizer during drug development. The data and trial protocol were kindly provided to us on request through the Vivli platform.

### Subjects

Healthy normal adult volunteers were recruited to a single investigational center in the United States. The volunteers were men and women aged between 18 and 55 years with normal clinical laboratory and electrocardiograph findings, no underlying illnesses, and who were not taking other medications.

### Ethics approval

The protocol and consent documents were reviewed and approved by the Institutional Review Board of the Pfizer Research Clinic. The study was conducted in accordance with the International Conference on Harmonization Guidelines for Good Clinical Practice, the Declaration of Helsinki, and in compliance with US Food and Drug Administration regulations for informed consent and protection of subject rights as described in 21 Code of Federal Regulations 50, 56, and 312. Written informed consent was obtained from all participants. Subjects were told that they were free to withdraw from the study at any time.

### Study design

This was a single‐center, open‐label, placebo‐controlled, randomized, parallel, 3‐day dosing study. The previously healthy adult volunteers were allocated to one of five different dosing regimens. Subjects were randomly assigned to treatment regimens according to a computer‐generated pseudo‐random code using the method of random permuted blocks.

1. Placebo, 2 placebo tablets per day for 3 days (*n* = 24).
2. Chloroquine phosphate 1,000 mg, equivalent to 600 mg chloroquine base (Aralen; Bayer, Myerstown, PA) daily for 3 days (*n* = 25).
3. Chloroquine phosphate 1,000 mg + azithromycin 500 mg (Zithromax; Pfizer, Ann Arbor, MI) daily for 3 days (*n* = 23).
4. Chloroquine phosphate 1,000 mg + 1,000 mg of azithromycin daily for 3 days (*n* = 24).
5. Chloroquine phosphate 1,000 mg + 1,500 mg of azithromycin daily for 3 days (*n* = 23).

Blood samples were taken at −1 (predose), 2, 6, 23, 26, 30, 47, 49, 50, 51, 52, 53, 54, 56, 58, 60, 72, 96, and 120 hours after the first dose. All participants received the drugs at 08:00 am daily for 3 days (days 0, 1, and 2) and the electrocardiograph (ECG) measurements (described below) were conducted in triplicate just before blood sampling. Subjects fasted overnight for 8 hours before study days −1, 0, 1, and 2, and continued to fast until after the 4‐hour timepoint.

### Bioanalytical methods

Serum concentrations of azithromycin were measured by a validated liquid chromatography/ electron capture method at BAS Analytics (West Lafayette, IN). The lower limit of quantitation was 10.4 ng/mL. The precision of the assay was within 5.9% and the accuracy ranged from −1.7% to 4.3%. Plasma concentrations of chloroquine and its metabolite desethylchloroquine were measured by a liquid chromatography/ mass spectrometry/mass spectrometry method at Bioassay Laboratory (Houston, TX). The lower limit of quantitation was 1 ng/mL for chloroquine and 0.5 ng/mL for desethylchloroquine. The precision of the assay was within 9.5% for chloroquine and 8.5% for desethylchloroquine and the accuracy ranged from −0.1% to 4.6% for chloroquine and −3.2 to 1.0% for desethylchloroquine.

### Pharmacokinetic analysis

Observed plasma chloroquine and azithromycin concentrations were logarithmically transformed and analyzed using nonlinear mixed‐effects modeling using NONMEM version 7.4 (Icon Development Solution, Ellicott City, MD). Pirana version 3.0.0,^20^ Perl‐speaks‐NONMEM version 4.8.0 (PsN),^21^ and R version 4.0.4 were used for automation, model evaluation, and diagnostics during the model building process.

All pharmacokinetic parameters were modeled as log‐normally distributed (Eq. 1).

(1) $$\Theta_{\mathrm{ij}}=\Theta \cdot \exp\left(\eta_{i,\Theta }+\kappa_{\mathrm{ij},\Theta }\right)$$

where $\Theta_{\mathrm{ij}}$ is the pharmacokinetic parameter for the ith individual on the jth occasion, Θ is the geometric mean parameter for the population, $\eta_{i,\Theta }$ is the interindividual variability (IIV) of the parameter Θ of the ith individual, and $\kappa_{\mathrm{ij},\Theta }$ is the interoccasion variability (IOV) of the parameter Θ of the ith individual on the jth occasion. The IIVs and IOVs were assumed to be normally distributed with a zero mean and a variance $\omega^{2}$. Pharmacokinetic parameters with an estimated IIV and IOV below 10% or with poor precision (%RSE > 50%) were fixed to zero.

Individual body‐weight (${\mathrm{BW}}_{i}$) was added into the pharmacokinetic model as an allometric function (i.e., the exponent of all volume parameters was fixed to 1.00 and the exponent of all clearance parameters was fixed to 0.75), scaled to the median body‐weight in the study (80.8 kg) as shown in Eq. 2.

(2) $$\Theta_{\mathrm{ij}}=\Theta \cdot \exp\left(\eta_{i,\Theta }+\kappa_{\mathrm{ij},\Theta }\right)\cdot {\left(\frac{{\mathrm{BW}}_{i}}{80.8}\right)}^{n}$$

All available covariates (age, sex, race, treatment arm, and height) were investigated by a stepwise addition (*P* < 0.05) and elimination (*P* < 0.001) approach.

Residual unexplained variabilities were modeled as additive errors on the log‐transformed observed plasma chloroquine and serum azithromycin concentrations. This is essentially equivalent to a proportional error on the arithmetic scale.

### Electrocardiographic analysis

ECG measurements were performed in triplicate 2 minutes apart and the mean of the 3 ECG interval measurements was used for the analysis to account for intrinsic variability. The machine read intervals were used in the analysis but these were manually checked to ensure correct QT reading. Where blood samples were taken at the same time, the ECG was completed before sampling (i.e., −1 (predose), 2, 6, 23, 26, 30, 47, 49, 50, 51, 52, 53, 54, 56, 58, 60, 72, 96, and 120 hours after the first dose). The baseline ECG was scheduled to be measured at the same clock times at 1 day before the drug administrations to account for diurnal variations. Baseline ECG measurements (at day −1) were used to determine the QT‐RR relationship by estimating the QT rate correction factor (α) according to Eq. 3.^22^

(3) $$\mathrm{QT}=QT_{c}\cdot RR^{\alpha }$$

Different correction factors were evaluated, including Bazzet’s formula (α=0.5), Fridericia’s formula (α=0.33), a population‐based optimal correction (estimated α from a regression analysis of QT vs. RR), and a subject‐specific individual correction (estimated individual $\alpha_{i}$’s). The baseline ECG data were also used to determine the heart rate dependence of the QRS and JT intervals. A linear correction factor was introduced to the QRS‐RR relationship as a population‐based and subject‐specific correction.^23^, ^24^ The JT‐RR relationship was evaluated in the same way as the rate correction of QT intervals (i.e., estimating a correction factor α). In addition, we evaluated a simpler way of correcting the JT interval by calculating the differences between corrected QT and QRS intervals, according to Eq. 4.^25^, ^26^

(4) JTc=QTc−QRS

ECG interval changes from the baseline measurement were assessed using a time‐matched analysis as suggested by the International Conference on Harmonization (ICH) E14 guideline.^27^, ^28^ The QTc prolongation (ΔQTc), QRS prolongation (ΔQRS), JTc prolongation (ΔJTc), and PR prolongation (ΔPR) were modeled separately. Effects of the administered drugs (chloroquine and azithromycin) were introduced into the pharmacodynamic model as a linear or maximum effect ($E_{\max}$) function (Eqs. 5, 6).

(5) $$\Delta \mathrm{QT}c_{\mathrm{ijk}}=\left(\Theta_{1}+\eta_{1,i}\right)+\Theta_{2}\cdot {\mathrm{TRT}}_{j}+\sum_{\text{Drug}}\left(\Theta_{3}+\eta_{3,i}\right)\cdot {\mathrm{CP}}_{\mathrm{ijk}}\;$$

(6) $$\Delta \mathrm{QT}c_{\mathrm{ijk}}=\left(\Theta_{1}+\eta_{1,i}\right)+\Theta_{2}\cdot {\mathrm{TRT}}_{j}+\sum_{\mathrm{Drug}}^{}\frac{\left(E_{\max}+\eta_{4,i}\right)\cdot {\mathrm{CP}}_{\mathrm{ijk}}}{{\mathrm{EC}}_{50}+{\mathrm{CP}}_{\mathrm{ijk}}}\;$$

where $\Delta \mathrm{QT}c_{\mathrm{ijk}}$ is the change from the time‐matched baseline in QTc for subject it in treatment j and at time k, $\Theta_{1}$ is the population mean intercept in the absence of treatment effect, $\eta_{1,i}$ is an interindividual random effect of the intercept term $\Theta_{1}$, $\Theta_{2}$ is the fixed effect associated with chloroquine treatment, ${\mathrm{TRT}}_{j}$ is the treatment arm (0 = placebo arm, 1 = all active treatment arms), $\Theta_{3}$ is a population mean slope of the linear function, $\eta_{3,i}$ is an interindividual random effect of the slope term $\Theta_{3}$, ${\mathrm{CP}}_{\mathrm{ijk}}$ is the observed plasma or serum drug concentrations, $E_{\max}$ is the maximum effect of the $E_{\max}$ function, $\eta_{4,i}$ is an interindividual random effect of the $E_{\max}$, and $EC_{50}$ is the plasma or serum concentration associated with 50% of the maximum effect. The estimated pharmacokinetic parameters were assumed to be normally distributed with a zero mean and $\omega^{2}$ variance.

The ΔPR, ΔQRS, and ΔJTc were also modeled as for the ΔQTc prolongation, using Eqs. 5 and 6.

Residual unexplained variabilities of ΔQTc, ΔQRS, ΔJTc, and ΔPR prolongation were modeled as an additive error.

### Model diagnostic and evaluations

The objective function value (OFV; calculated by NONMEM as proportional to −2 × log‐likelihood of the data) was used as a proxy of overall model fit. A likelihood ratio test was used to discriminate between any two hierarchical models (i.e., a reduction of OFV more than 3.84 is equivalent to a *P* value < 0.05 at 1 degree of freedom difference. Basic goodness‐of‐fit plots were used to determine potential model misspecification and systematic errors. Eta and epsilon shrinkages were used to assess the ability to detect model misspecifications in goodness‐of‐fit diagnostics.^29^ Model robustness and parameter confidence intervals (CIs) were evaluated by a sampling‐important‐resampling procedure.^30^, ^31^ Predictive performances of the final models were illustrated by prediction corrected visual and numerical predictive checks (*n* = 2,000).^32^ The 5th, 50th, and 95th percentiles of the observed concentrations were overlaid with the 95% CIs of each simulated percentile to detect model bias.

Placebo‐adjusted ΔΔQTc was computed based on the final pharmacometric model according to a methodology suggested by Garnett *et al*.^28^ The change from baseline QTc adjusted for placebo effect (ΔΔQTc) is defined as the difference between the model‐driven ΔQTc at the concentration of interest (C) and the model‐driven ΔQTc for placebo (concentration = 0).

(8) $$\begin{array}{c} \;\text{mean}\Delta \Delta \text{QTc}\left(C\right)=\text{mean}\;\left(\;\Delta \mathrm{QT}c_{\mathrm{ijk}}\;|\;j=1,{\mathrm{CP}}_{\mathrm{ijk}}=C\;\right)\\ \;-\text{mean}\;\left(\;\Delta \mathrm{QT}c_{\mathrm{ijk}}\;|\;\;j=0,{\mathrm{CP}}_{\mathrm{ijk}}=0\;\right) \end{array}$$

Median and 90% CIs of the placebo‐adjusted ΔΔQTc we computed using a non‐parametric bootstrap method (*n* = 1,000 resampled dataset), stratified by study arm. The model parameters and $\text{mean}\Delta \Delta \text{QTc}\left(C\right)$ were calculated from each of the replicate bootstrapped datasets. Two‐sided 90% CIs were determined from the 5th and 95th percentile of the ranked‐ordered $\Delta \Delta \text{QTc}\left(C\right)$ values from all of the bootstrapped datasets.

The placebo‐adjusted PR prolongation (ΔΔPR), placebo‐adjusted QRS prolongation (ΔΔQRS), and placebo‐adjusted JTc prolongation (ΔΔJTc) were also modeled as for the ΔΔQTc prolongation.

### Code and data availability

Data used in this study are from the data contributors Pfizer, Ltd. who conducted the volunteer pharmacometrics study. This has been made available through Vivli (https://Vivli.org). All relevant NONMEM codes for the pharmacokinetic and pharmacodynamic models are available from the authors upon request and are also freely available at the DDMoRe Model Repository (http://repository.ddmore.eu/models).

## RESULTS

This study recruited 119 healthy volunteers who were randomized into the 5 treatment arms. Baseline characteristics of the volunteers are summarized in the **Table** **S1**.

### Chloroquine pharmacokinetics

**Figure 1:**
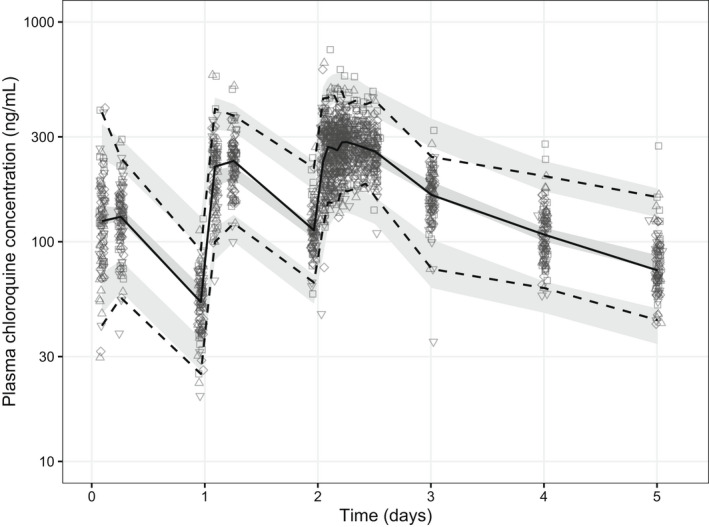
Visual predictive plots of the final pharmacokinetic model of plasma chloroquine concentrations. Solid and dashed lines represent the median, 5th, and 95th percentiles of the observations. Shaded areas represent the predictive 95% confidence intervals of each percentile.

Chloroquine plasma concentrations were modeled as a two‐compartment distribution model. A three‐compartment distribution model did not improve the model fit because the observation period was only 5 days. A transit‐compartment model did not improve the absorption model significantly, but the parallel absorption model did improve the model fit (ΔOFV = −9.43). IIVs and IOVs were introduced to the absorption parameters. Stepwise covariate modeling found that the inter‐compartment clearance (Q_1_/F) was reduced according to the dosing occasion (ΔOFV = −234). No other covariate was statistically significant. Importantly, co‐administration with azithromycin did not affect chloroquine pharmacokinetics. The final model estimated the pharmacokinetic parameters precisely (**Table** **1**), fitted the data well (**Figure** **S1**), and had good predictive performance (**Figures** **1**, **S2**).

**Table 1:** Parameter estimates of the final pharmacokinetic model of chloroquine and azithromycin

Pharmacokinetic parameter	Chloroquine pharmacokinetics		Azithromycin pharmacokinetics	
	Typical value^a^ (%RSE^b^)	95% CI^b^	Typical value^a^ (%RSE^b^)	95% CI^b^
Population parameter estimate				
F (%)	100% (*fixed*)	NA	100% (*fixed*)	NA
K_a1_ (hr^−1^)	0.350	0.273, 0.449	1.59 (12.4%)	1.42, 2.30
K_a2_ (hr^−1^)	0.169 (17.6%)	0.112, 0.221	NA	NA
CL/F (L∙hr^−1^)	47.4 (4.46%)	43.0, 51.4	101 (3.61%)	94.4, 108
V_C_/F (L)	2,550 (3.29%)	2,380, 2,720	451 (8.57%)	402, 563
Q_1_/F (L∙hr^−1^)	347 (6.85%)	304, 394	80.6 (5.01%)	73.7, 89.4
V_P1_/F (L)	6,480 (5.62%)	5,830, 7,260	2,510 (4.17%)	2,340, 2,740
Q_2_/F (L∙hr^−1^)	NA	NA	343 (6.49%)	304, 394
V_P2_/F (L)	NA	NA	824 (5.46%)	714, 883
OCC on Q_1_/F	−0.286 (1.71%)	−0.295, −0.275	NA	NA
Inter‐individual variability/Inter‐occasion variability* (%CV)				
F (%)	16.7% (9.54%)/14.1%* (10.3%)	14.0%, 20.2%/11.5%, 17.0%*	21.5% (8.52%)/14.9%* (9.89%)	18.0%, 25.3%/12.0%, 17.7%*
K_a1_ (hr^−1^)	73.3% (15.1%)/71.0%* (14.1%)	53.5%, 109%/51.9%, 99.0%*	59.4% (25.5%)/51.0%* (13.7%)	28.3%, 63.9%/46.2%, 90.3%*
CL/F (L∙hr^−1^)	18.5% (7.87%)	15.3%, 20.9%	14.8% (9.78%)	13.0%, 19.1%
V_C_/F (L)	11.4% (14.4%)	7.73%, 14.1%	49.6%* (10.6%)	37.6%, 58.2%*
Q_1_/F (L∙hr^−1^)	32.6%* (11.1%)	25.2%, 40.6%*	NA	NA
V_P1_/F (L)	18.2%* (13.9%)	12.9%, 22.7%*	NA	NA
Q_2_/F (L∙hr^−1^)	NA	NA	30.1% (12.8%)	24.8%, 40.3%
Unexplained residual error				
σ	0.0178 (4.71%)	0.0163, 0.0194	0.0194 (5.09%)	0.0179, 0.0218

The * indicate inter‐occasion variability (%CV). %CV, percent coefficient of variation; CI, confidence interval; CL/F, oral clearance; F, relative bioavailability; K_a1_, first‐order absorption rate constant; K_a2_, paralleled first‐order absorption rate constant; NA, not applicable; OCC, dosing occasion; V_C_/F, central apparent volume of distribution; V_P1_/F, first peripheral compartment apparent volume of distribution; V_P2_/F, second peripheral compartment apparent volume of distribution; Q_1_/F, first inter‐compartment clearance; Q_2_/F, second inter‐compartment clearance; RSE, relative standard error; σ, variance of unexplained residual error.

^a^ Computed population mean parameter estimates from NONMEM were calculated for a typical patient at a body weight of 80.8 kg. The %CV of the inter‐individual variability (IIV) and inter‐occasion variability (IOV) was calculated as $100\times \sqrt{\exp\left(\omega^{2}\right)-1}$.

^b^ Computed from the sampling‐important‐resampling (SIR) procedure^30^, ^31^ of the final pharmacokinetic model with five iterations of 1000, 1000, 1000, 2000, and 2000 number of the proposal sampling and 200, 200, 400, 500, and 500 number of resampling.

### Azithromycin pharmacokinetics

**Figure 2:**
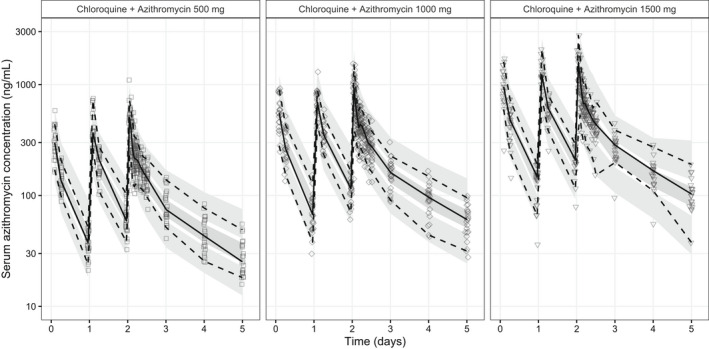
Visual predictive plots of the final pharmacokinetic model of plasma azithromycin concentrations, stratified by treatment arm. Solid and dashed lines represent the median, 5th, and 95th percentiles of the observations. Shaded areas represent the predictive 95% confidence intervals of each percentile.

Azithromycin serum concentrations were modeled as a three‐compartment distribution model. The addition of transit‐compartments did not improve the absorption model, so the single first‐order absorption model was retained in the final model. IIVs and IOVs were also introduced to the absorption parameters. No covariate improved the population pharmacokinetic model significantly. The final pharmacokinetic model estimated the pharmacokinetic parameters precisely (**Table** **1**), fitted the data well (**Figure** **S3**), and had good predictive performance (**Figure** **2**).

### Electrocardiographic effects

There was a small but significant increase in the heart rate in the 4 hours after chloroquine administration which was not affected by azithromycin co‐administration; mean (SD) heart rate of placebo arm: 57.5 (6.2)/minute compared with 64.8 (7.8)/minute in the chloroquine arms (*P* < 0.0001). There was also a small but significant prolongation of the PR interval (atrioventricular conduction time) at 5 hours after chloroquine dosing; mean (SD) PR interval in the placebo arm: 166.7 (29.7) ms vs. 173.6 (20.8) in the chloroquine arms (*P* < 0.0001). The mean (SD; range) prolongation was 6.22% (6.18; −13.5 to 33.3). One subject in the placebo group and one subject in the chloroquine dosing group had a PR interval of more than 220 ms.

### Heart‐rate corrected electrocardiographic intervals

The individual baseline QT and RR intervals were highly correlated. Four heart rate correction factors, applied to the baseline electrocardiographic data, were compared: Fridericia’s (QT/RR^0.33^), Bazett’s (QT/RR^0.50^), a population‐based correction (QT/RR^α^), and a subject‐specific individual correction ($\mathrm{QT}/{\mathrm{RR}}^{\alpha_{i}}$) (**Figure** **S4**). The population‐based correction factor (α) was estimated as 0.360 (95% CI: 0.347 to 0.374). As expected, subject‐specific heart rate‐corrected QT intervals (estimated to 0.376 (95% CI: 0.211 to 0.553)) resulted in the least correlation with heart rate. Even though the baseline QRS and RR intervals exhibited a slight linear trend, the mean subject‐specific correction slope was estimated to be −0.000402 (95% CI: −0.00827 to 0.00823; **Figure** **S5**). Heart rate correction of the QRS interval was therefore unnecessary. The JT interval was evaluated independently in the same way as the QT interval (i.e., estimated subject‐specific correction factor). However, as the QRS interval was rate independent, the JTc interval derived from the simpler method (i.e., JTc = QTc − QRS) resulted in very similar values as the subject‐specific JT correction (**Figure** **S6**). Therefore, for parsimony, the corrected JT intervals were derived from the QTc and QRS intervals. These correction methods were applied for each subject’s subsequent ECG observations.

### Chloroquine and azithromycin effects on electrocardiographic changes

**Figure 3:**
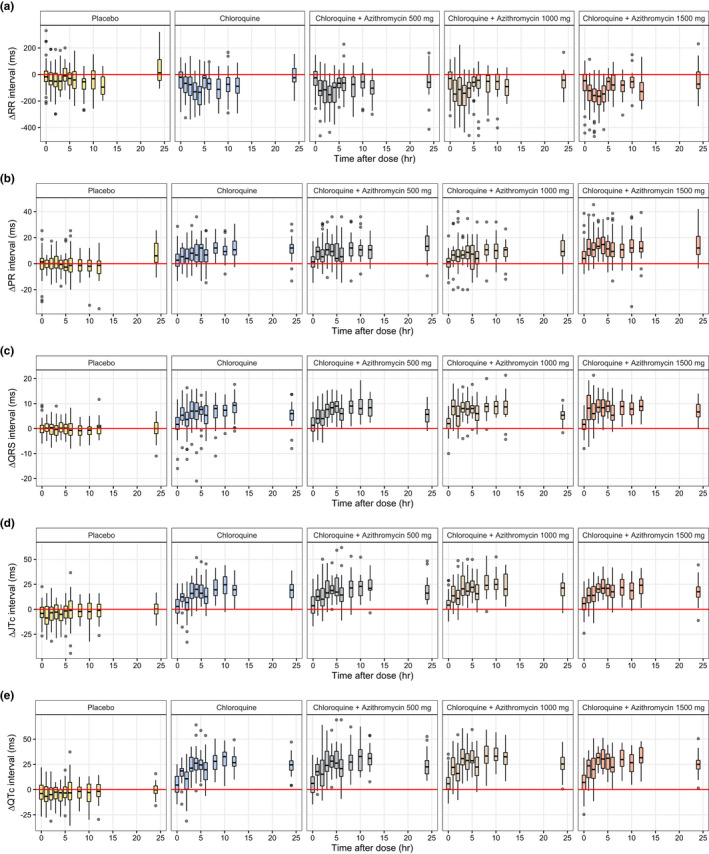
Electrocardiographic changes after the last day of drug administration, stratified by treatment arm. (**a**) ΔRR intervals, (**b**) ΔPR intervals, (**c**) ΔQRS intervals, (**d**) ΔJTc intervals, and (**e**) ΔQTc intervals vs. time after dose (c refers to subject‐specific individual heart rate‐correction).

**Figure 4:**
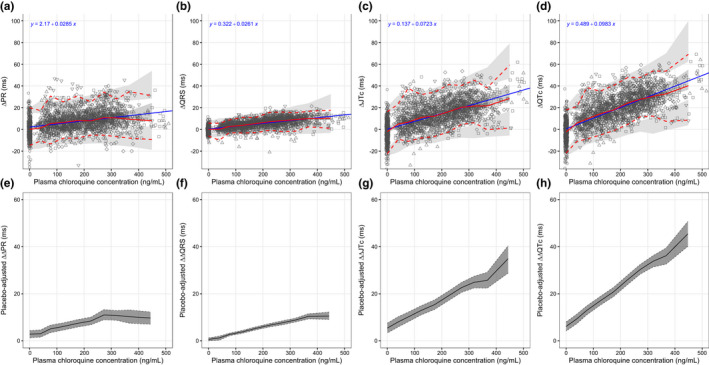
Visual predictive plot of the relationship between the prolongation of electrocardiographic intervals and the corresponding plasma chloroquine concentrations (**a–d**) and placebo‐adjusted electrocardiographic intervals for the corresponding plasma chloroquine concentrations (**e–h**): ΔPR (**a**), ΔQRS (**b**), ΔJTc (**c**), ΔQTc (**d**) ΔΔPR (**e**), ΔΔQRS (**f**), ΔΔJTc (**g**), and ΔΔQTc (**h**). Solid and dashed red lines represent the median, 5th, and 95th percentiles of the observations. Solid blue lines and equations show the simple linear regression model fit. Shaded areas in panel **a–d** represent the 95% confidence intervals (CIs) of predictions at each percentile, based on the final population pharmacodynamic model. Solid lines and shaded areas in panel **e–h** represent the median placebo‐adjusted electrocardiographic intervals and 90% CIs.

**Figure 5:**
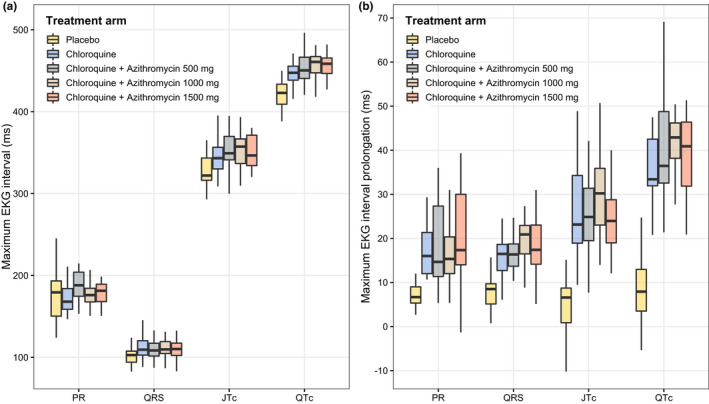
Boxplot of the maximum electrocardiographic intervals (**a**) and the maximum electrocardiographic prolongations (**b**) between each treatment arm. The JT, and QT intervals have been individually heart‐rate corrected. The mean (SD; range) of the maximum changes on PR, QRS, JTc, and QTc intervals in all chloroquine administration arms are 18.4 (8.80; −1.33 to 39.33) ms, 12.1 (3.55; 3.66 to 21.3) ms, 30.9 (10.8; −2.02 to 61.8) ms, and 38.8 (11.4; 6.66 to 69.11) ms, respectively.

The ECG changes (ΔRR, ΔPR, ΔQRS, ΔQTc, and ΔJTc intervals) were calculated from the difference in the electrocardiographic interval measurements before and after the treatment using time‐matched data in order to adjust for the circadian rhythm effect (**Figures** **3** **,** **S7** **, and** **S8**).^27^ The ECG changes (ΔPR, ΔQRS, ΔJTc, and ΔQTc) were modeled using a linear and $E_{\max}$ relationships with the observed plasma chloroquine and serum azithromycin concentrations. The $E_{\max}$ was not superior to the linear model, and was not retained moving forward. The parameter estimates for the final ECG interval model were estimated precisely (**Table** **2**). The goodness‐of‐fit of the final model is illustrated in **Figures** **S9–S12**. The visual predictive check plots for all models of ΔPR, ΔQRS, ΔJTc, and ΔQTc were satisfactory (**Figures** **4** **,** **S13–S16**). The placebo‐adjusted ΔΔPR, ΔΔQRS, ΔΔJTc, and ΔΔQTc are presented in **Figures** **4** and **S17–S20**. Chloroquine produced concentration‐dependent QRS interval prolongation. The mean (SD; range) maximum QRS prolongation following 1,000 mg of chloroquine phosphate (600 mg base) was 12.1 (3.55; 3.66 to 21.3) ms. This corresponds to a mean (SD; range) increase of 13.5% (4.15%; 3.78 to 24.5%). JT interval prolongation was also concentration‐dependent. The mean (SD; range) maximum JT prolongation following 1,000 mg of chloroquine phosphate (600 mg base) was 30.9 (10.8; −2.02 to 61.8) ms. This corresponded to a mean (SD; range) increase of 9.72% (3.42%; −0.620 to 18.9%). As a result of the prolongation of these two intervals (which added together provide the QT interval), the QT interval prolongation was also concentration‐dependent. The mean (SD; range) maximum QT prolongation following 1,000 mg of chloroquine phosphate (600 mg base) was 38.8 (11.4; 6.66 to 69.1) ms. This corresponded to a mean (SD; range) increase of 9.45% (2.80%; 1.53 to 16.2%; **Figures** **5** **and** **S21**). Chloroquine prolonged the QTc interval by ~ 9.75 ms per 100 ng/mL, of which 28.3% resulted from QRS widening and 71.1% from JTc prolongation. Azithromycin exhibited a minimal effect on these ECG effects when co‐administered with chloroquine (**Figure** **5**). The time course of each effect was tightly correlated, although maximal values did not always coincide.

**Table 2:** Parameter estimates of the final pharmacodynamic model of ΔPR, ΔQRS, ΔJTc, and ΔQTc intervals

Pharmacodynamic parameter	ΔPR		ΔQRS		ΔJTc		ΔQTc	
	Typical value^a^ (%RSE^b^)	95% CI^b^	Typical value^a^ (%RSE^b^)	95% CI^b^	Typical value^a^ (%RSE^b^)	95% CI^b^	Typical value^a^ (%RSE^b^)	95% CI^b^
Population parameter estimate								
Placebo baseline (ms)	0.424 (34.9%)	0.260, 0.808	−0.151 (46.9%)	−0.283, 0.0179	−2.42 (23.1%)	−3.10, −1.14	−2.57 (19.7%)	−3.31, −1.46
Baseline (ms)	2.87 (23.4%)	1.47, 4.14	0.646 (25.4%)	0.309, 0.964	5.46 (16.1%)	3.61, 6.97	6.15 (12.5%)	4.62, 7.60
Chloroquine effect (ms per 100 ng/mL)	2.77 (9.90%)	2.23, 3.30	2.76 (4.89%)	2.51, 3.03	7.03 (4.70%)	6.36, 7.70	9.75 (3.58%)	9.03, 10.5
Azithromycin effect (ms per 100 ng/mL)	−0.110 (35.1%)	−0.138, 0.0144	−0.110 (34.8%)	−0.186, −0.0359	−0.68 (14.5%)	−0.876, −0.489	−0.800 (14.8%)	−1.05, −0.57
Inter‐individual variability (%CV)								
Placebo baseline (ms)	4.22% (9.60%)	3.50%, 5.05%	1.41% (11.3%)	1.11%, 1.75%	4.53% (8.27%)	3.89%, 5.33%	4.40% (9.28%)	3.65%, 5.25%
Chloroquine effect (ms per 100 ng/mL)	1.92% (16.0%)	1.59%, 2.24%	1.00% (9.64%)	0.817%, 1.20%	1.95% (10.2%)	1.56%, 2.31%	2.26% (9.64%)	1.80%, 2.66%
Azithromycin effects (ms per 100 ng/mL)	0.316% (9.00%)	0.201%, 0.403%	0.316% (13.9%)	0.230%, 0.401%	0.361% (20.6%)	0.187%, 0.470%	0.548% (10.1%)	0.445%, 0.655%
Unexplained residual error								
σ_ADD_ (ms)	7.34% (3.13%)	7.15%, 7.59%	2.98% (3.33%)	2.90%, 3.10%	8.92% (3.22%)	8.67%, 9.23%	9.13% (3.30%)	8.85%, 9.44%

CI, confidence interval; RSE, relative standard error; σ_ADD_, additive unexplained residual error.

^a^ The interindividual variabilities of the pharmacodynamic model were assumed to be normally distributed with the coefficient of variation (%CV) of $100\times \sqrt{\omega^{2}}$.

^b^ Computed from the sampling‐important‐resampling (SIR) procedure^30^, ^31^ of the final pharmacokinetic model with five iterations of 1000, 1000, 1000, 2000, and 2000 number of the proposal sampling and 200, 200, 400, 500, and 500 number of resampling.

## DISCUSSION

The pharmacokinetic properties of oral chloroquine and azithromycin in healthy volunteers were approximately similar to those reported previously. The observed plasma chloroquine concentrations were characterized adequately by a two‐compartment model. This did not capture the long terminal half‐life of 1–2 months.^7^, ^33^ However, peak concentrations are associated with maximum ECG effects and these relevant early high plasma concentrations of chloroquine were captured by the sampling schedule and described accurately by the developed model. Slow absorption of chloroquine was modeled as two parallel first‐order processes (K_a1_ = 0.350 hr^−1^ and K_a2_ = 0.169 hr^−1^). Both rate and extent of the absorption process were found to be highly variable. Azithromycin pharmacokinetics were well‐characterized by a three‐compartment model. Azithromycin has a shorter terminal half‐life than chloroquine of 2–4 days.^34^ Peak concentrations of azithromycin occurred 1–2 hours after the dose. For both drugs, the pharmacokinetic estimates were similar to those reported previously in healthy volunteers. There was no evidence for pharmacokinetic interaction between the two drugs, as reported previously.^35^, ^36^

Chloroquine delayed atrioventricular and intraventricular conduction and also delayed ventricular repolarization in proportion to the plasma concentrations.^7^, ^37^, ^38^ Although macrolides are associated with QT prolongation, despite the high doses evaluated, the addition of azithromycin had very little effect on the chloroquine‐induced changes. Similar findings were reported in an individual comparison conducted in patients with COVID‐19.^39^ The highest azithromycin doses tested in this study (4.5 g over 3 days) are higher than those used in COVID treatment and so reinforce the lack of significant electrophysiological effects from azithromycin. The 4‐aminoquinolines block several different myocardial cation channels. In voltage‐clamped cat ventricular myocytes exposed to chloroquine, the order of inhibitory potencies from greatest to least was inward rectifying potassium current (IK1), rapid delayed rectifying potassium current (IKr), inward sodium current (INa), and L‐type calcium current (ICa‐L). Chloroquine (and the closely related hydroxychloroquine) blocks the rapid component but not the slow component of the delayed rectifying outward current.^40^, ^41^ Chloroquine also blocks the hyperpolarization‐activated funny current (*If*), which plays a major role in the sino‐atrial node pacemaker and may cause bradycardia, although, in this study, there was a consistent small concentration‐dependent increase in heart rate.^42^ Blockade of the inwardly rectifying potassium (hERG) channel, which delays ventricular repolarization and thus prolongs the QT interval, has been the focus of most concern.^10^, ^11^, ^12^ The hERG channel blockade is a risk factor for polymorphic ventricular tachycardia (TdP), although there is much debate about the determinants of the risk relationship, and the potential ameliorating effect of multichannel blockade. In general, the multichannel block caused by chloroquine and hydroxychloroquine has been considered “unbalanced” and, therefore, a risk factor for TdP.^38^ Until the definitive large randomized trials showed clearly that it was not beneficial in patients hospitalized with COVID‐19,^5^, ^6^ hydroxychloroquine was the most widely used COVID‐19 treatment in the world. So great was the recent concern over ECG QT prolongation that QT prolongation comprised the majority of reported “adverse effects” associated with chloroquine and hydroxychloroquine in the prevention and treatment of COVID‐19, and it was the main reason for prematurely terminating treatment.

There has also been debate over the measurement of the QT interval, and the relative merits of manual vs. machine reading, and uncertainty over the optimal rate correction for the QT interval. The Fridericia correction (1/RR^0.33^) has been preferred for healthy subjects, whereas the Bazett correction (1/RR^0.50^) often performs better in sick patients.^43^ This study was sufficiently large and detailed that individual corrections could be derived to avoid any confounding by heart rate. In addition, the circadian rhythm effects on ECG measurements were accounted for by modeling (**Figure** **S5**) time‐matched differences. Most clinical reports on hydroxychloroquine and chloroquine in COVID‐19 prevention and treatment describe the QT interval changes, but they seldom report the other ECG intervals. The QT interval comprises the sum of ventricular depolarization and ventricular repolarization times. These two fundamentally different electrophysiological processes have very different implications for cardiotoxicity. Indeed, in chloroquine overdose, it is the QRS widening (intraventricular conduction delay) that has a greater prognostic value.^44^ A major finding of this study is that over one quarter (28.3%) of the observed QT prolongation caused by chloroquine resulted from QRS widening (ventricular depolarization). Nearly all the recent reports and viewpoints cautioning against hydroxychloroquine potential cardiotoxicity (i.e., QT prolongation) have not distinguished between QRS and JT prolongation. Thus, if this same ratio of QRS to JT prolongation observed in this study applies in patients with COVID‐19,^37^ then the degree of drug‐induced repolarization delay (and corresponding assumed tachyarrhythmia risk) has been overestimated substantially.

Myocardial involvement is common in severe COVID‐19. Arrhythmias occurred in approximately one‐sixth of hospitalized patients during the early phase of the pandemic.^45^, ^46^ QT prolongation may occur independent of drug treatment in severe COVID‐19. This confounds the interpretation of individual cases or observational series without appropriate controls. Valid causal interpretations can be derived reliably from randomized controlled trials and there are now a sufficient number of these to conclude that the use of hydroxychloroquine and chloroquine is not associated with a significantly increased risk of TdP (**Table** **2**). The exception was a trial from Brazil, which evaluated a substantially higher dose of chloroquine than other trials (10 mg base/kg twice daily for 10 days in one treatment arm).^47^ This trial was stopped early because 39% (16 of 41 patients) in the high‐dosage group died, in 2 cases preceded by ventricular tachycardia (not TdP), compared with 15% (6 of 40) in the lower dose arm. Whether or not this resulted from iatrogenic cardiotoxicity cannot be determined. The two largest randomized studies in hospitalized patients (RECOVERY and SOLIDARITY)^5^, ^6^ evaluated hydroxychloroquine at an adult maintenance dose of 400 mg/day for 10 days after an initial loading dose of 800 mg given twice. Although mortality was slightly higher in the hydroxychloroquine arms, the survival curves diverged after the end of treatment and there was no evidence for cardiotoxicity; no excess of cardiac arrhythmias, and no differences during the acute loading dose or at 10 days when plasma concentrations would have been highest. Large randomized controlled trials with lower hydroxychloroquine dose regimens in post‐exposure and pre‐exposure prophylaxis have enrolled over 10,000 patients. None of these have reported arrhythmias.^4^ Overall, there is no indication of a pro‐arrhythmic or anti‐arrhythmic effect in COVID‐19. A very large and carefully controlled observational study of over 900,000 patients with rheumatological disease starting hydroxychloroquine suggested that hydroxychloroquine reduces arrhythmias, and did not affect short term (30 days) mortality, but that adding azithromycin to chloroquine was associated with an increased risk of 30‐day cardiovascular mortality, angina, and heart failure—although the mechanism was unclear.^48^

Limitations of this study are that this was a three‐dose evaluation so any effects from longer term administration would not have been observed. The long terminal elimination phase of chloroquine was not captured by this sampling schedule, but the two‐compartment disposition model was sufficient to describe the observed plasma concentrations adequately. Although the electrocardiographic interval effects were tightly correlated, the maximal effects did not always coincide so the sum of the mean maximal reported values for QRS and JT prolongation exceed the maximal QT prolongation value. Uncertainty in QT measurements, particularly in the presence of T‐wave morphology changes associated with strong hERG block, could also contribute to imprecise estimates. This was a healthy volunteer study so any disease interaction, such as myocardial involvement in severe COVID‐19, could not be evaluated.

## CONCLUSIONS

Population pharmacokinetics and chloroquine concentration‐dependent ECG effects in healthy volunteers with chloroquine and azithromycin administration were characterized accurately in this large open‐label randomized healthy volunteer study. Plasma chloroquine and serum azithromycin concentrations were explained satisfactorily by two‐ and three‐compartment disposition models, respectively. There was no pharmacokinetic interaction between the drugs. Chloroquine caused concentration‐dependent delays in atrioventricular (PR interval) and intraventricular depolarization (QRS interval) and repolarization (JTc interval). Over one quarter of the QTc prolongation resulted from QRS prolongation, which suggests that the arrhythmogenic risk inferred from QT prolongation measurement alone has been overestimated substantially.

## FUNDING

This work was support by the Wellcome Trust (220211); the Bill and Melinda Gates Foundation (INV‐006052); and was part of the Wellcome Trust‐Mahidol University‐Oxford Tropical Medicine Research Programme. The funding bodies did not have any role in the collection, analysis, interpretation of data, writing of the manuscript, or in the decision in submitting the manuscript for publication. A Creative Commons Attribution 4.0 Generic License has already been assigned to the Author Accepted Manuscript version.

## CONFLICT OF INTEREST

The authors declared no competing interests for this work.

### AUTHOR CONTRIBUTIONS

All authors wrote the manuscript. N.J.W. and J.A.W. designed the research; P.C., J.T., and R.M.H. performed the research. P.C. analyzed the data.

## Supporting information


**Appendix S1** XXXX
