# Supplementary material for: Pharmacometric and Electrocardiographic Evaluation of Chloroquine and Azithromycin in Healthy Volunteers
Source: Clin Pharmacol Ther. 2022 Jun 22;112(4):824–35. doi: 10.1002/cpt.2665 (PMC9540484; doi:10.1002/cpt.2665)
Supplement: Supplementary file 1 — Appendix S1 XXXX [file CPT-112-824-s001.pdf]

Supplementary materials

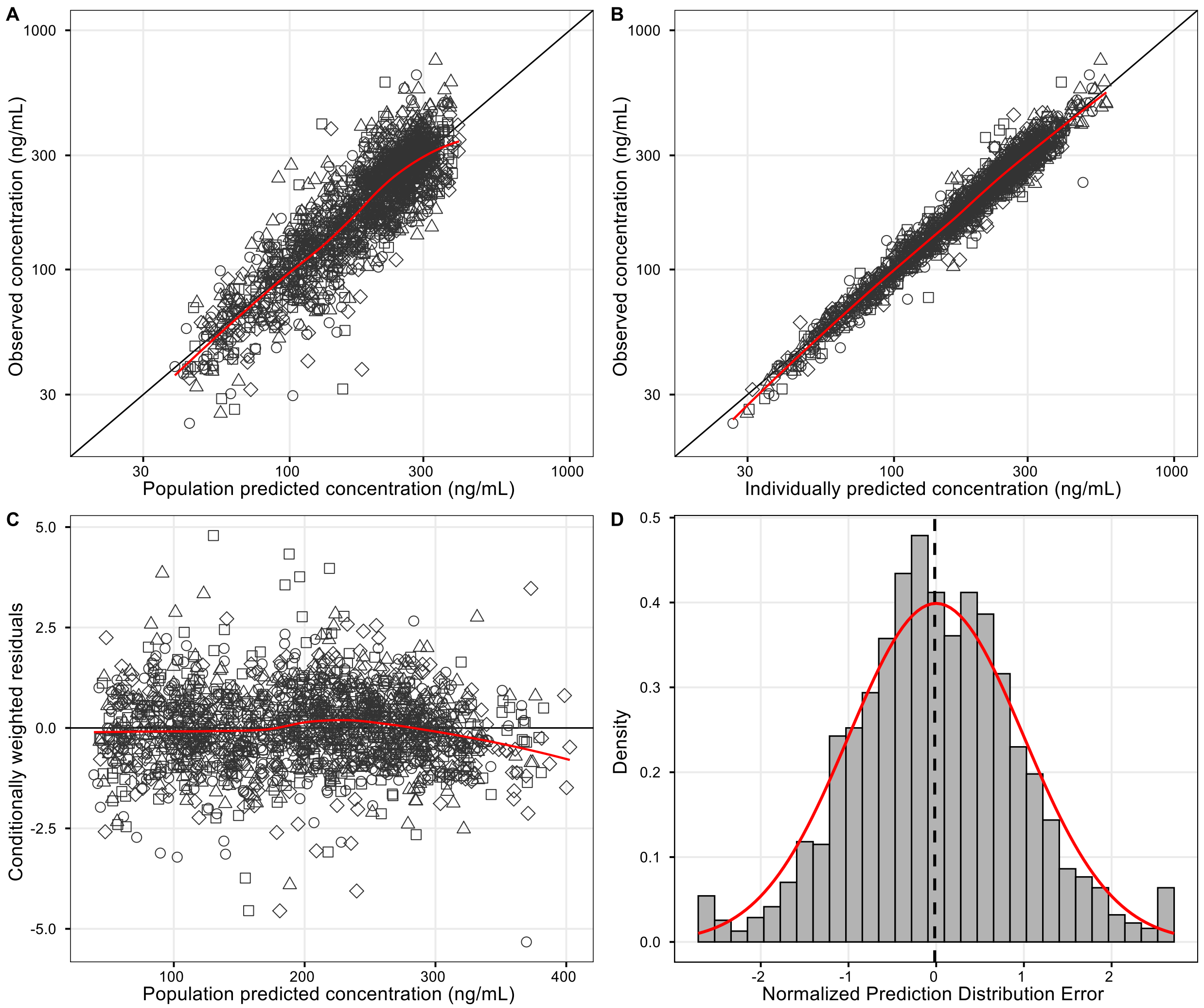

Figure S1: Goodness-of-fit plots for the final chloroquine population pharmacokinetic model. (A) Population predictions versus observations, (B) individual predictions versus observations, (C) population predictions versus conditionally weighted residuals, and (D) histogram of the normalized prediction distribution error (NPDE). Red lines represent the locally weighted least-square regression fits based on the observed concentrations.

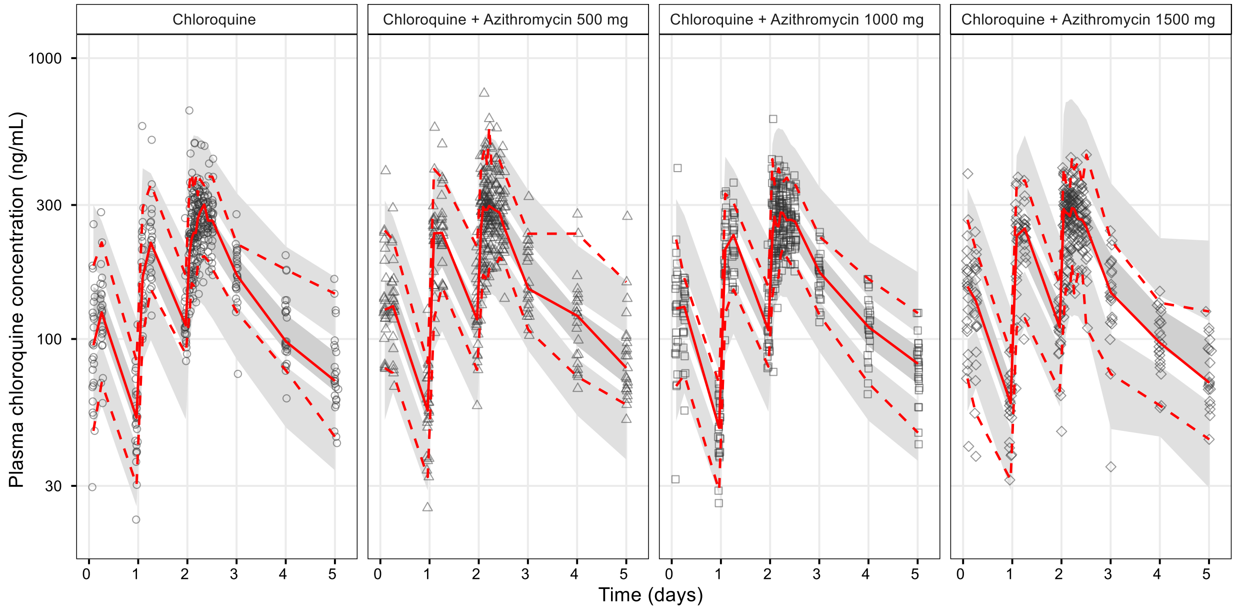

Figure S2: Visual predictive plots of the final chloroquine pharmacokinetic model, stratified by treatment arm. Solid and dashed lines represent the median, 5th, and 95th percentile of the observations. Shaded areas represent the predictive 95% confidence interval of each percentile.

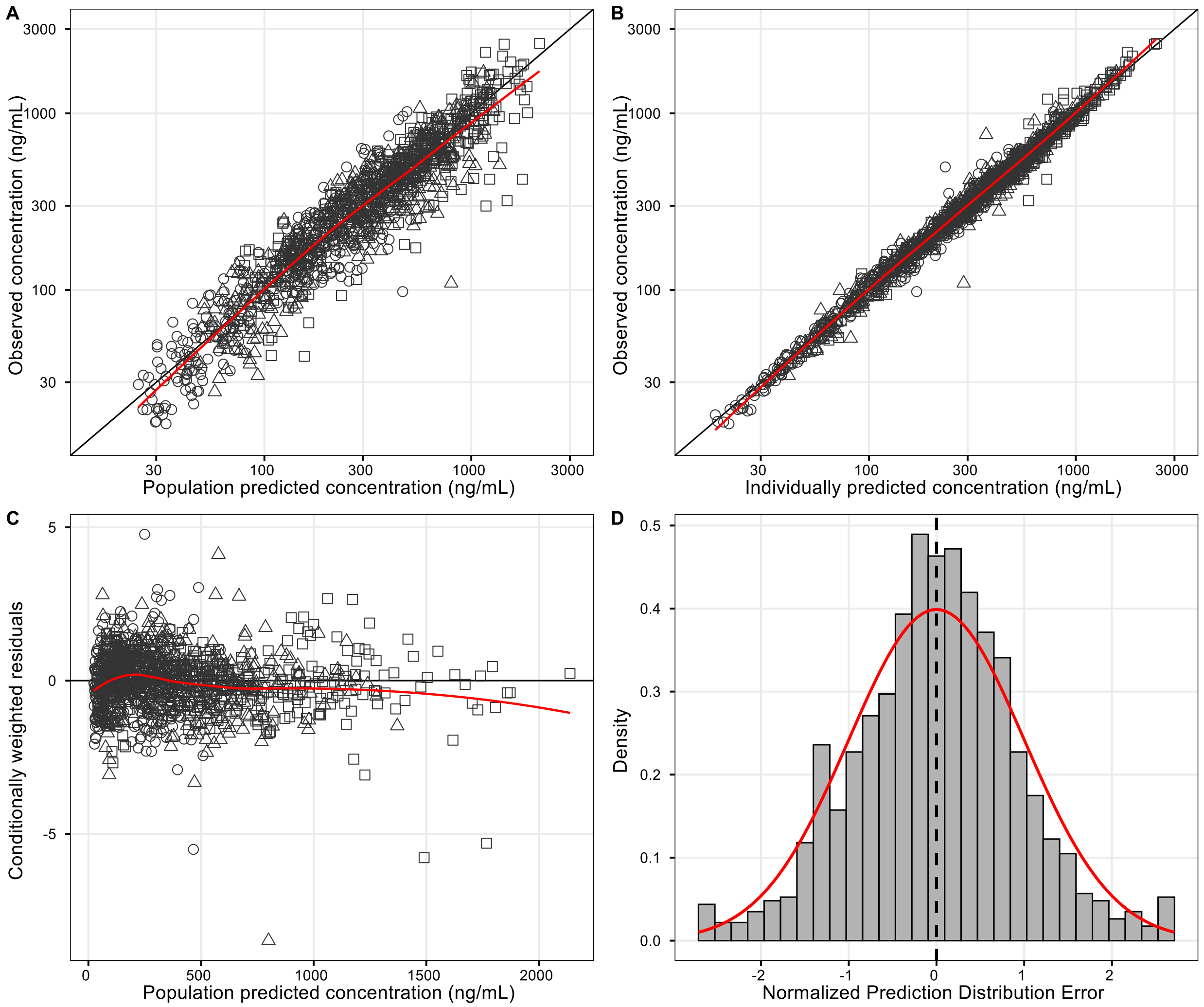

Figure S3: Goodness-of-fit plots for the final azithromycin population pharmacokinetic model. (A) Population predictions versus observations, (B) individual predictions versus observations, (C) population predictions versus conditionally weighted residuals, and (D) histogram of the normalized prediction distribution error (NPDE). Red lines represent the locally weighted least-square regression fits based on the observed concentrations.

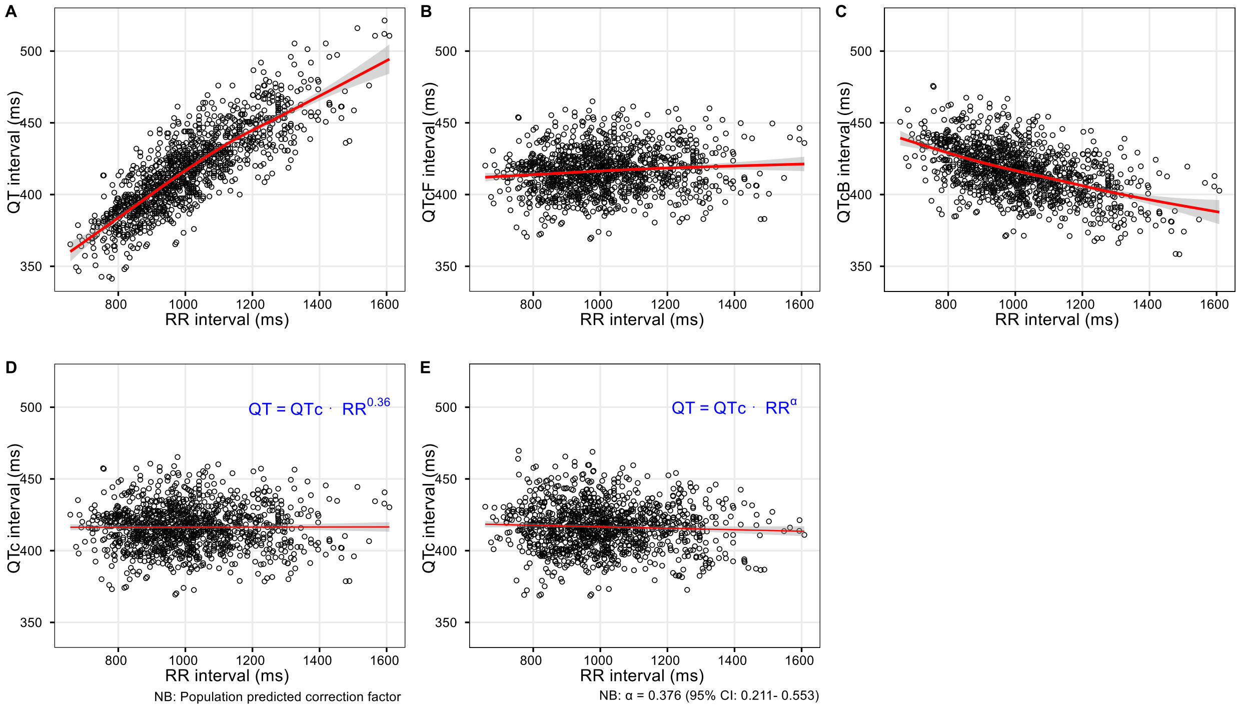

Figure S4: Rate corrected QT interval versus RR interval using different correction methods: (A) uncorrected, (B) using Fridericia's formula, (C) using Bazett's formula, (D) using an estimated population-based correction factor, and (E) using an estimated subject-specific individual correction factor. EKG measurements from all subjects were taken on the day before drug administration.

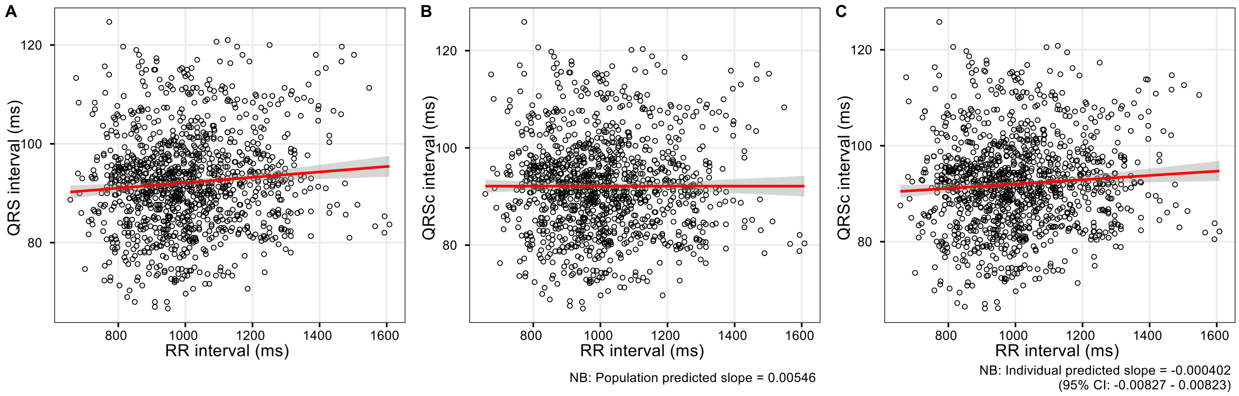

Figure S5: Rate corrected QRS interval versus RR interval using different correction methods: (A) uncorrected, (B) using an estimated population-based linear correction factor, and (C) using an estimated subject-specific individual linear correction factor. EKG measurements from all subjects were taken on the day before drug administration.

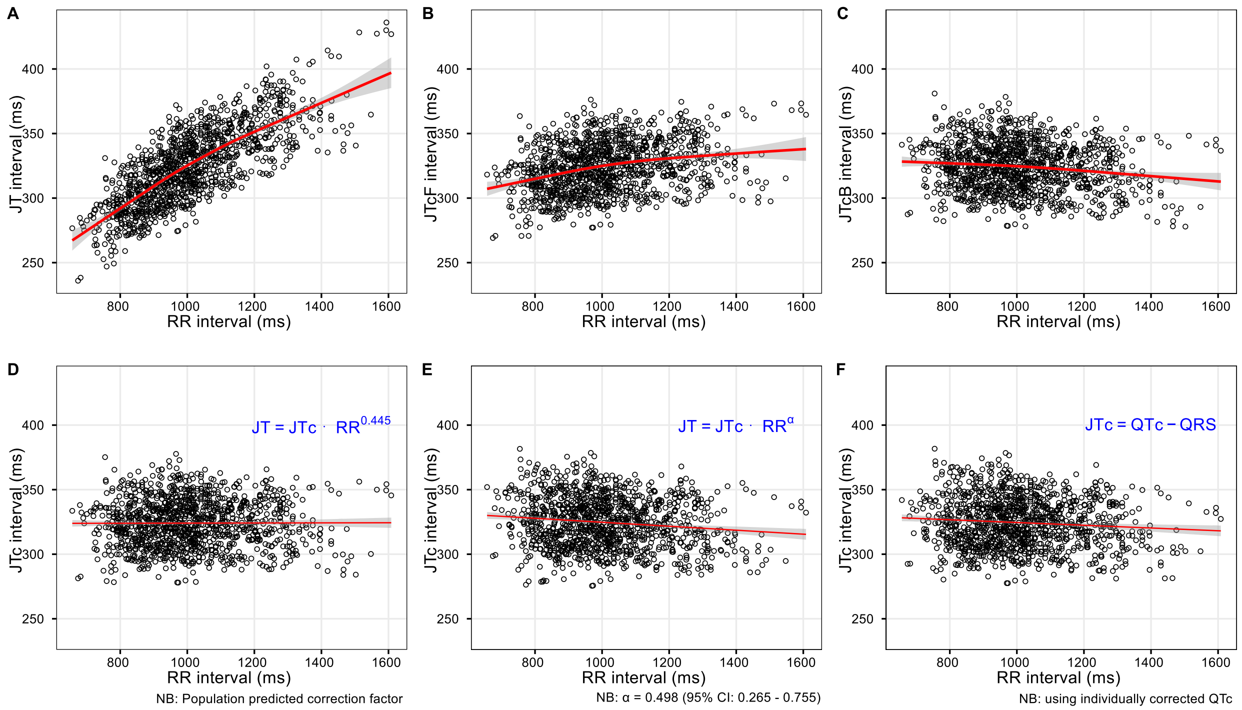

Figure S6: Rate corrected JT interval versus RR interval using different correction methods: (A) uncorrected, (B) using Fridericia's formula, (C) using Bazett's formula, (D) using an estimated population-based correction factor, (E) using an estimated subject-specific individual correction factor, and (F) using an estimated subject-specific JTc, based on QTc and QRS intervals. EKG measurements from all subjects were taken on the day before drug administration.

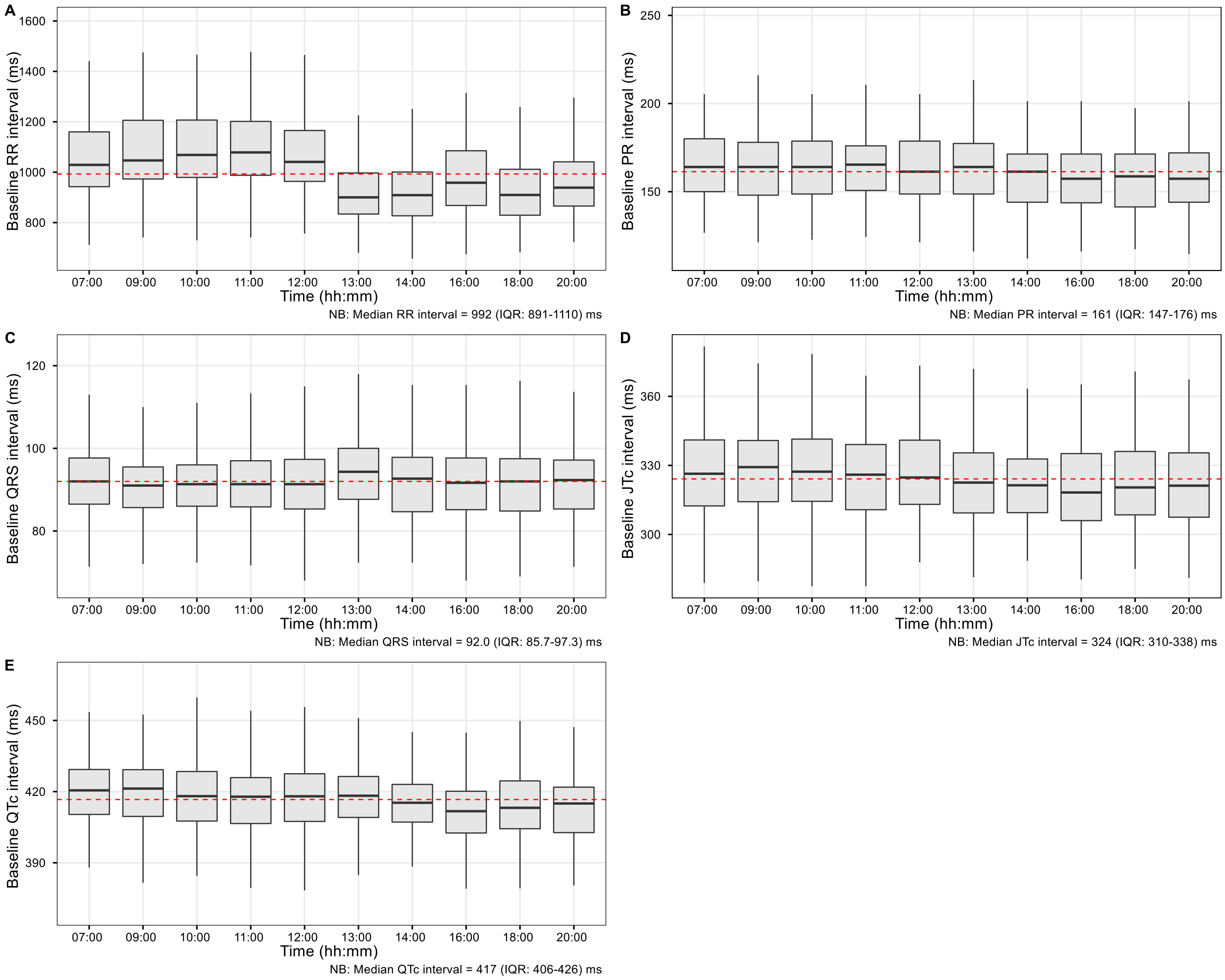

Figure S7: Baseline electrocardiographic measurements one day before drug administration. (A) RR intervals, (B) PR intervals, (C) QRS intervals, (D) JTc intervals, and (E) QTc intervals according to clock time. Electrocardiographic interval measurements exhibit a circadian rhythm, especially before and after 12:00 pm. Horizontal dashed lines represent the overall median electrocardiographic measurements.

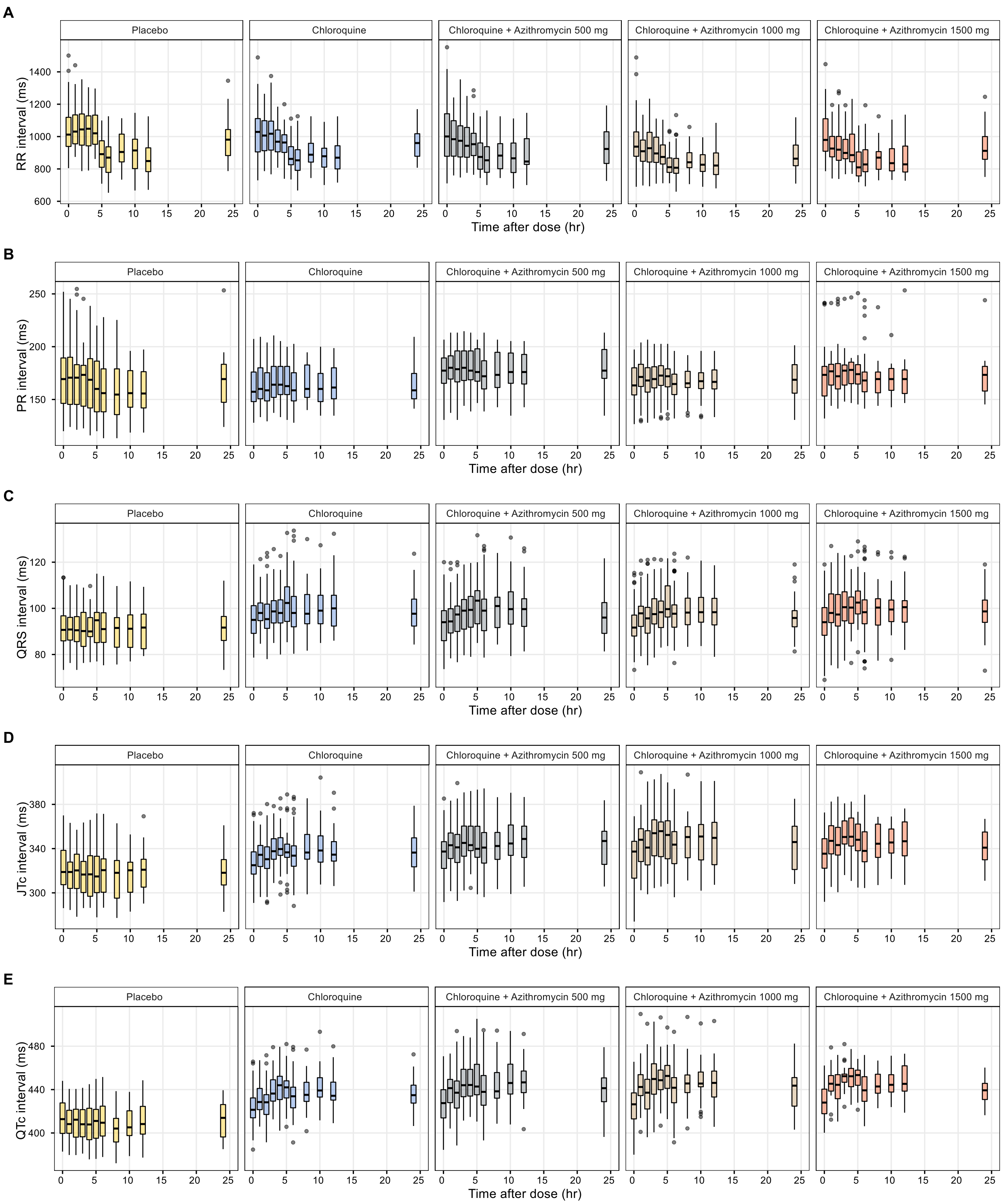

Figure S8: Electrocardiographic intervals after the last day of drug administration, stratified by treatment arm. (A) RR intervals, (B) PR intervals, (C) QRS intervals, (D) JTc intervals, and (E) QTc intervals versus time after dose (c refers to subject-specific individual heart rate-correction).

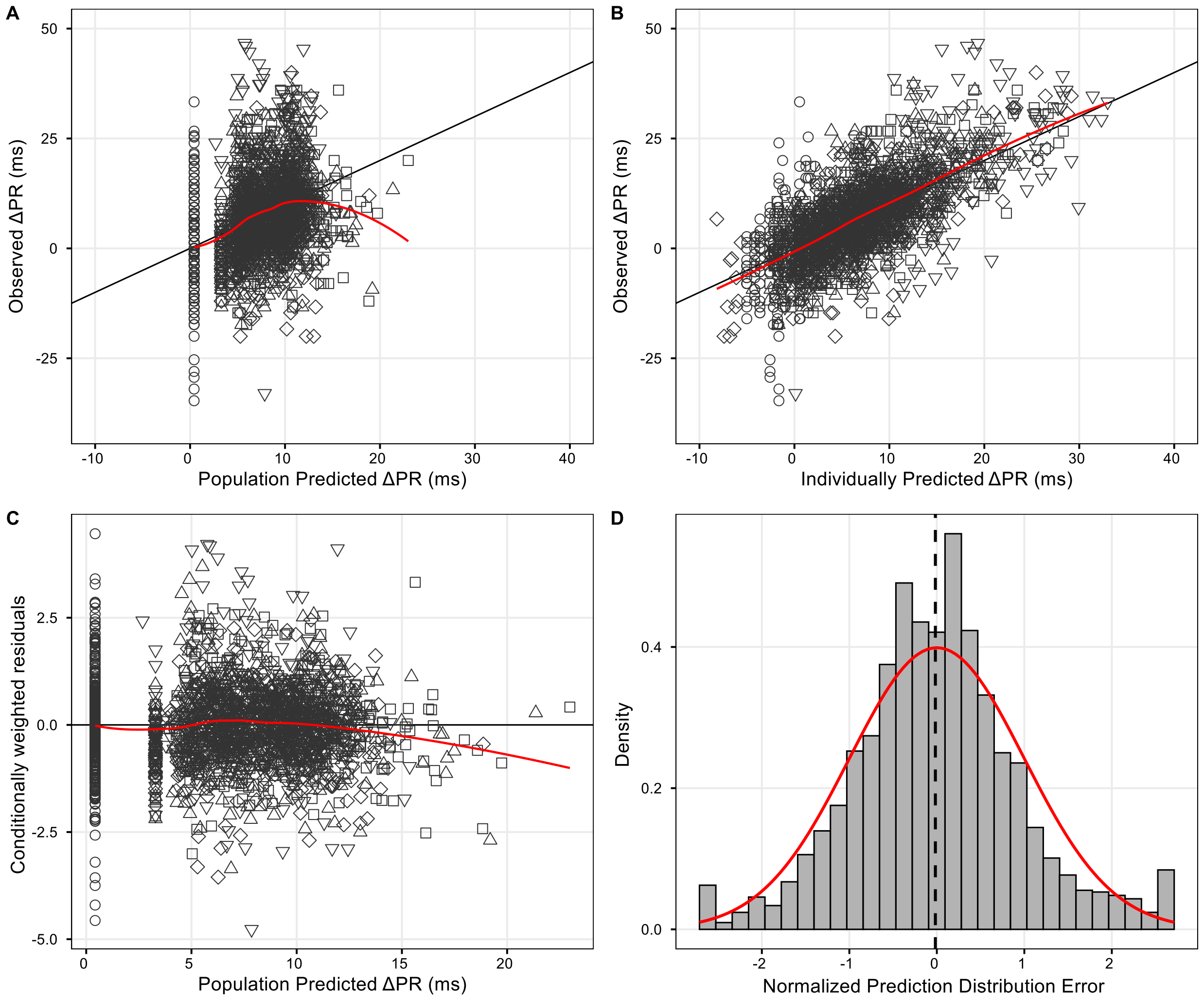

Figure S9: Goodness-of-fit plots for the final ΔPR model. (A) Population predictions versus observations, (B) individual predictions versus observations, (C) population predictions versus conditionally weighted residuals, and (D) histogram of the normalized prediction distribution error (NPDE). Red lines represent the locally weighted least-square regression fits based on the observed concentrations.

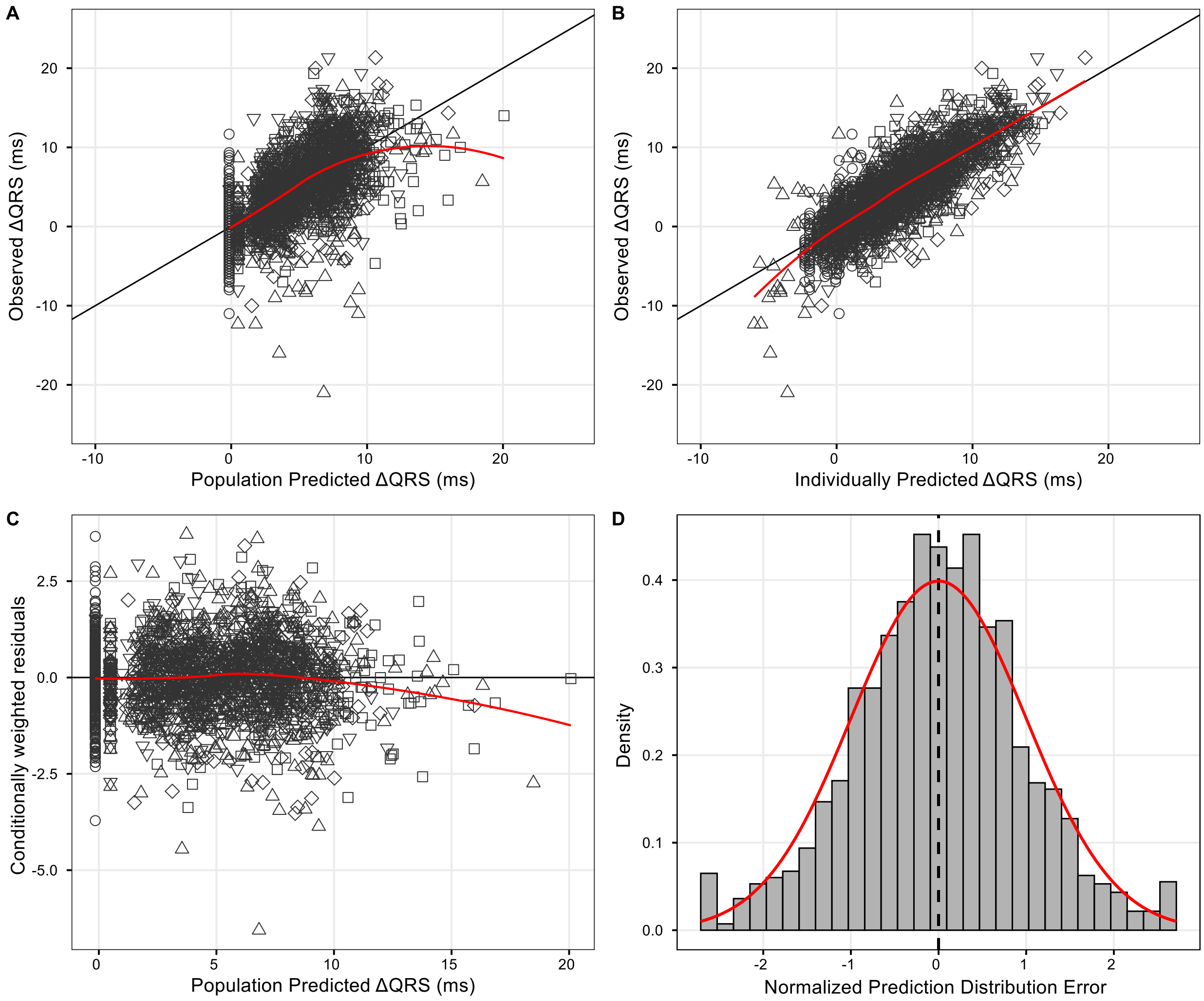

Figure S10: Goodness-of-fit plots for the final ΔQRS model. (A) Population predictions versus observations, (B) individual predictions versus observations, (C) population predictions versus conditionally weighted residuals, and (D) histogram of the normalized prediction distribution error (NPDE). Red lines represent the locally weighted least-square regression fits based on the observed concentrations.

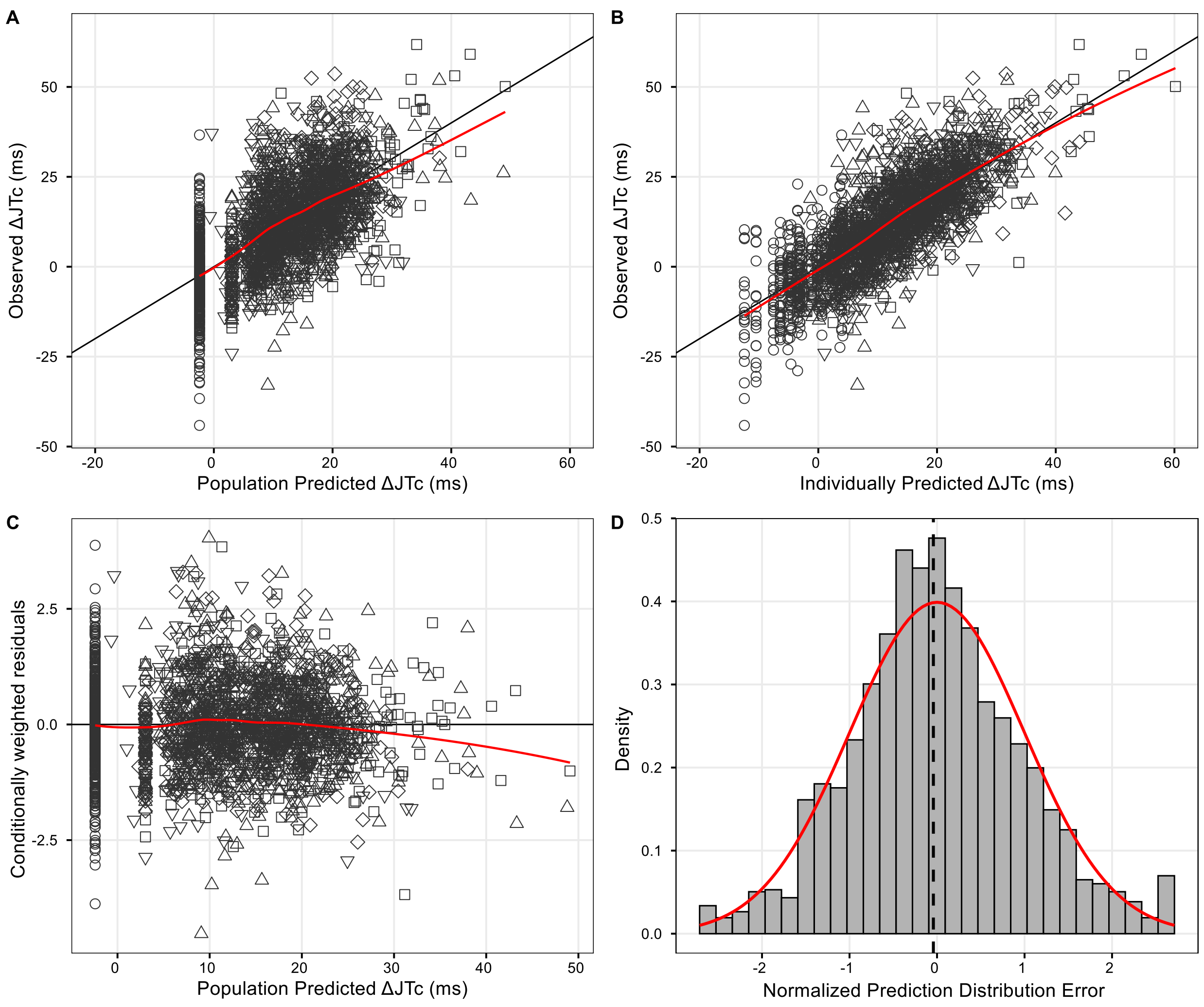

Figure S11: Goodness-of-fit plots for the final ΔJTc model. (A) Population predictions versus observations, (B) individual predictions versus observations, (C) population predictions versus conditionally weighted residuals, and (D) histogram of the normalized prediction distribution error (NPDE). Red lines represent the locally weighted least-square regression fits based on the observed concentrations.

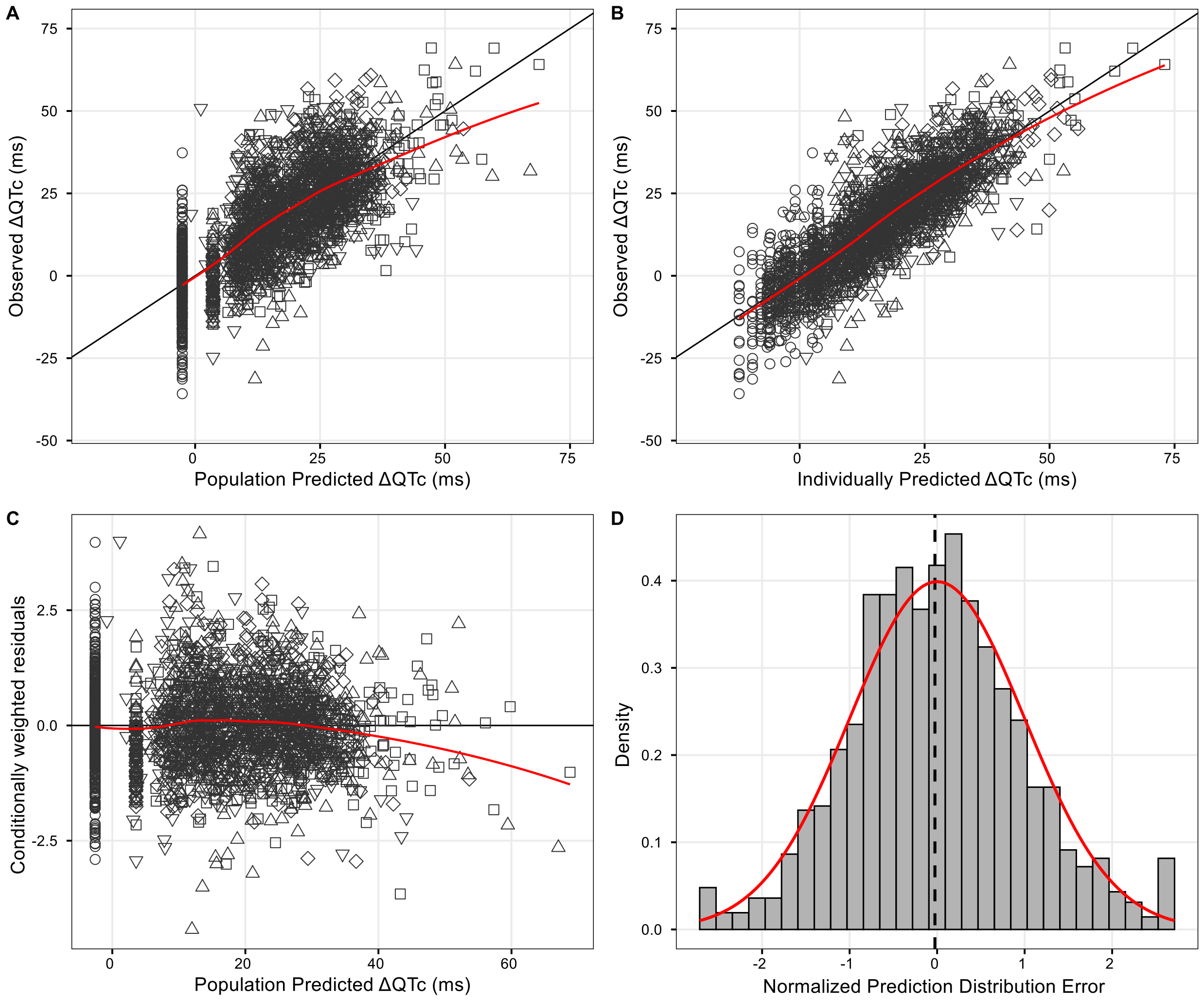

Figure S12: Goodness-of-fit plots for the final ΔQTc model. (A) Population predictions versus observations, (B) individual predictions versus observations, (C) population predictions versus conditionally weighted residuals, and (D) histogram of the normalized prediction distribution error (NPDE). Red lines represent the locally weighted least-square regression fits based on the observed concentrations.

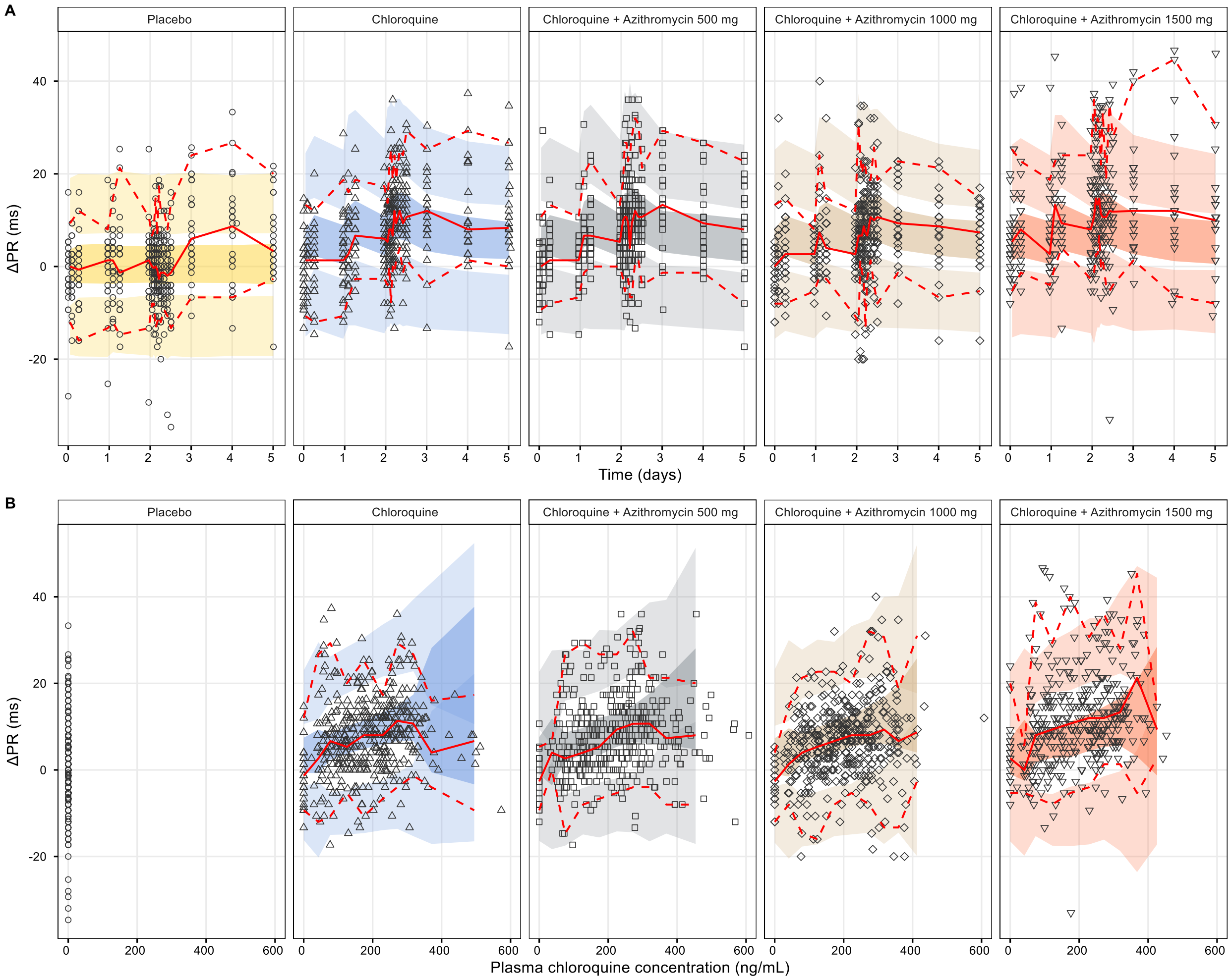

Figure S13: Visual predictive plot for the population PR model, stratified by treatment arm; (A) ΔPR interval vs. time after the first dose and (B) ΔPR interval vs. observed plasma chloroquine concentrations. Solid and dashed lines represent the median, 5th, and 95th percentiles of the observations. Shaded areas represent the predictive 95% confidence interval of each percentile.

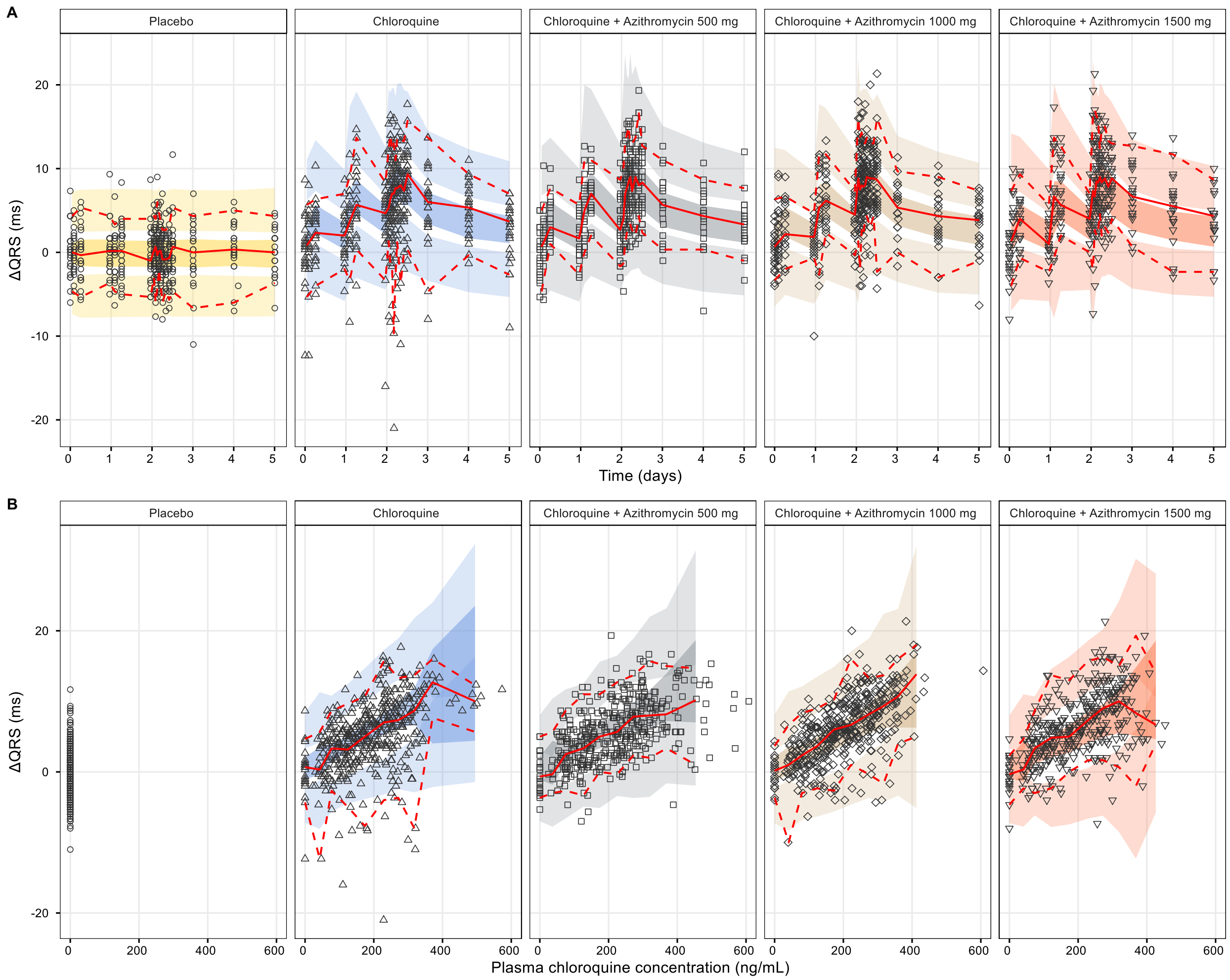

Figure S14: Visual predictive plot for the population QRS model, stratified by treatment arm; (A) ΔQRS interval vs. time after the first dose and (B) ΔQRS interval vs. observed plasma chloroquine concentrations. Solid and dashed lines represent the median, 5th, and 95th percentiles of the observations. Shaded areas represent the predictive 95% confidence interval of each percentile.

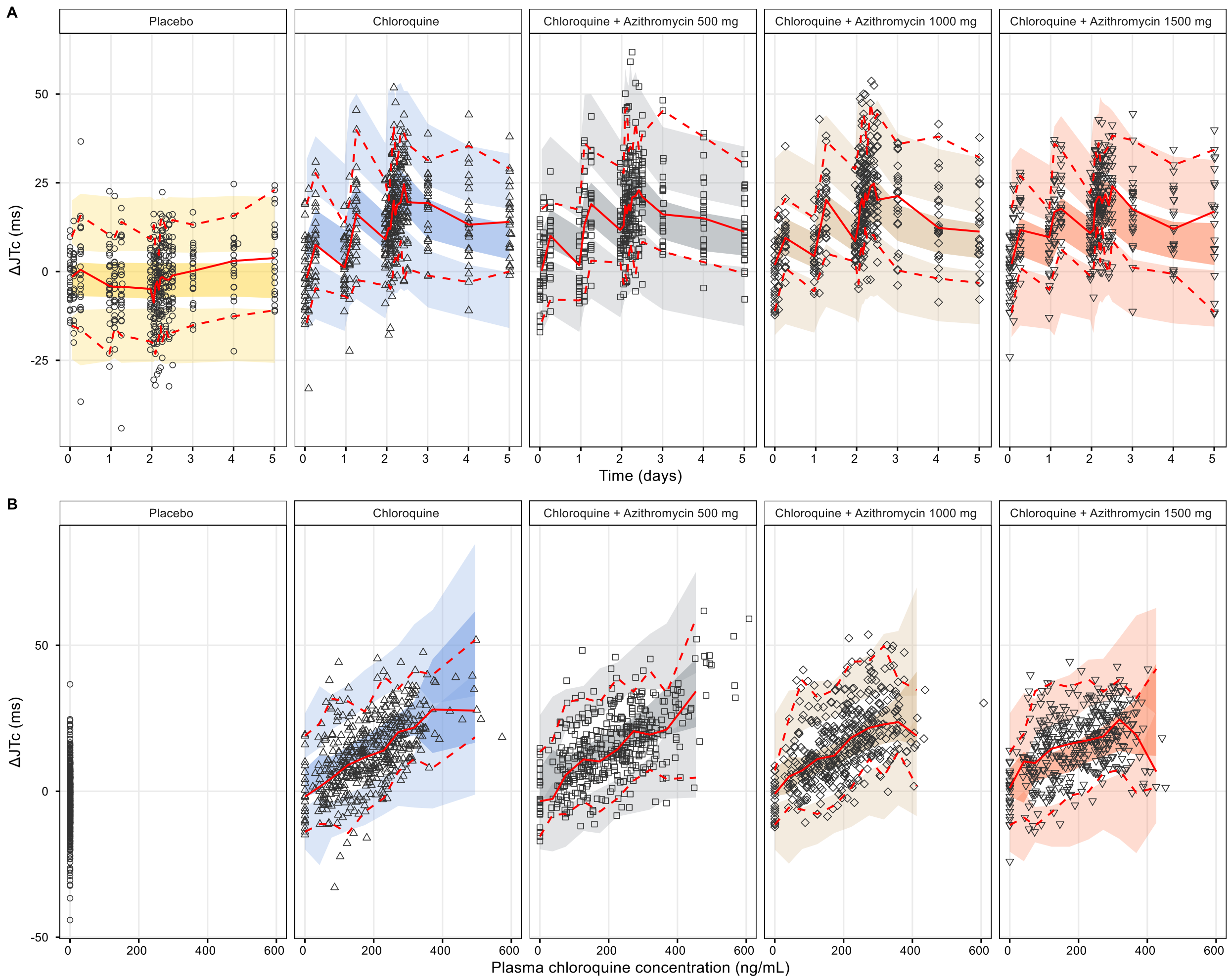

Figure S15: Visual predictive plot for the population JTc model, stratified by treatment arm; (A) ΔJTc interval vs. time after the first dose and (B) ΔJTc interval vs. observed plasma chloroquine concentrations. Solid and dashed lines represent the median, 5th, and 95th percentiles of the observations. Shaded areas represent the predictive 95% confidence interval of each percentile.

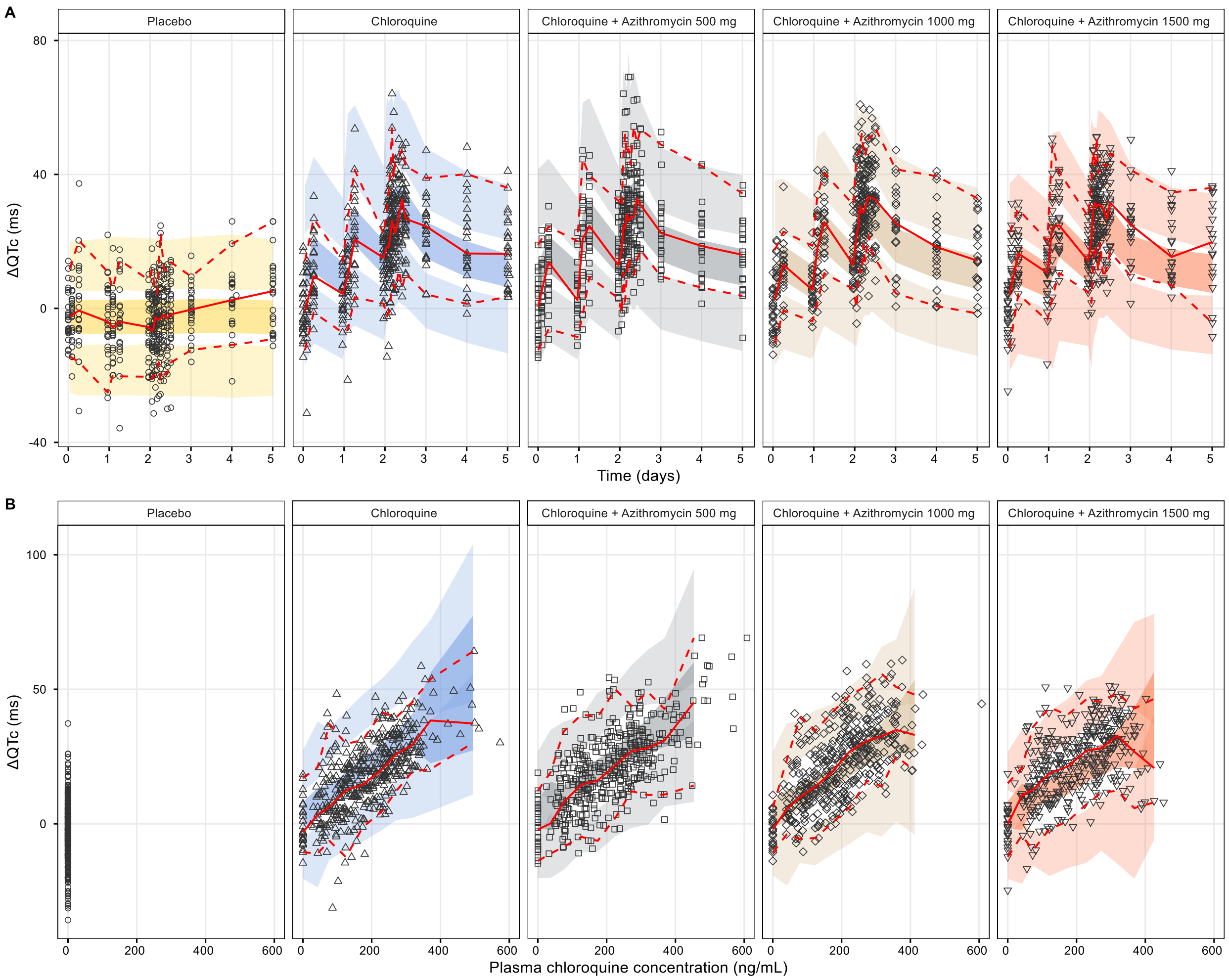

Figure S16: Visual predictive plot for the population QTc model, stratified by treatment arm; (A) ΔQTc interval vs. time after the first dose and (B) ΔQTc interval vs. observed plasma chloroquine concentrations. Solid and dashed lines represent the median, 5th, and 95th percentiles of the observations. Shaded areas represent the predictive 95% confidence interval of each percentile.

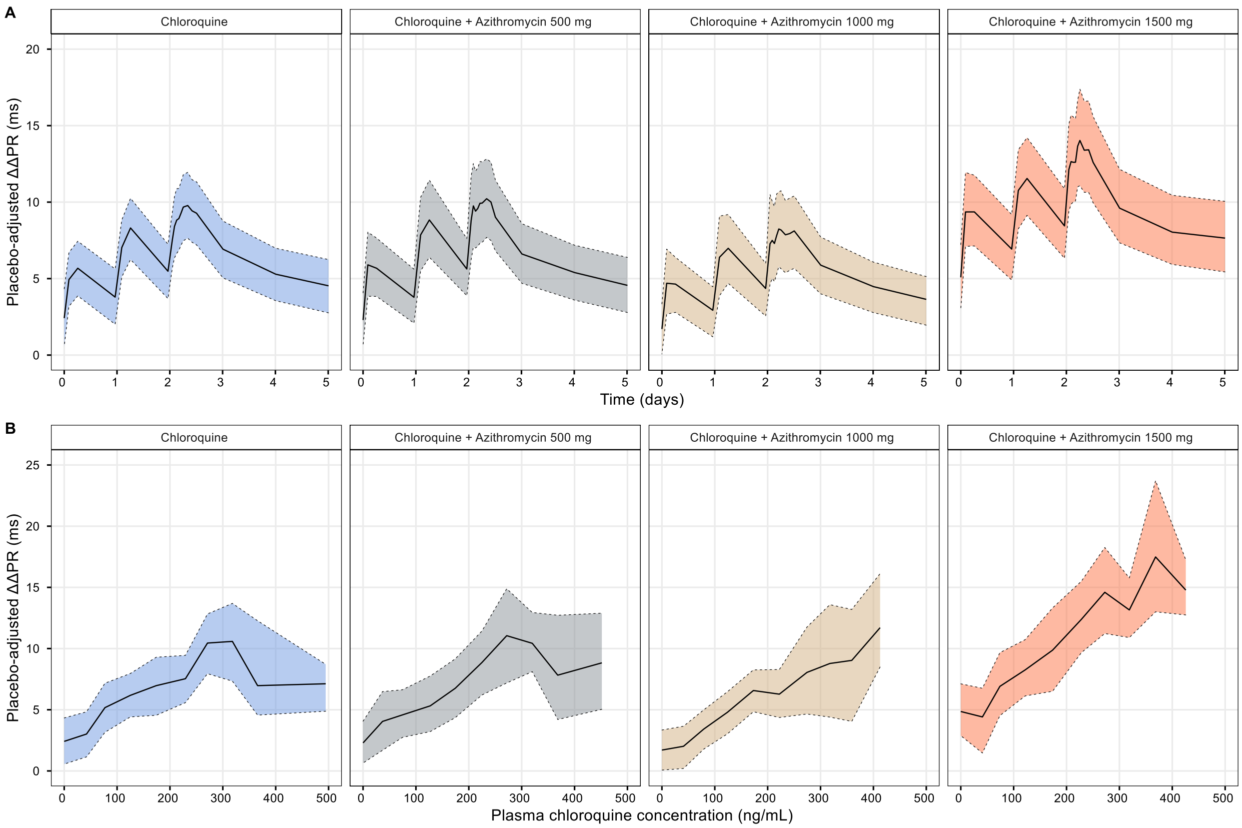

Figure S17: Placebo-adjusted ΔΔPR interval, stratified by treatment arms; (A) ΔΔPR interval vs. time after the first dose and (B) ΔΔPR interval vs. observed plasma chloroquine concentrations. Solid lines represent the predicted median ΔΔPR interval. Shaded areas represent the predicted 90% confidence interval.

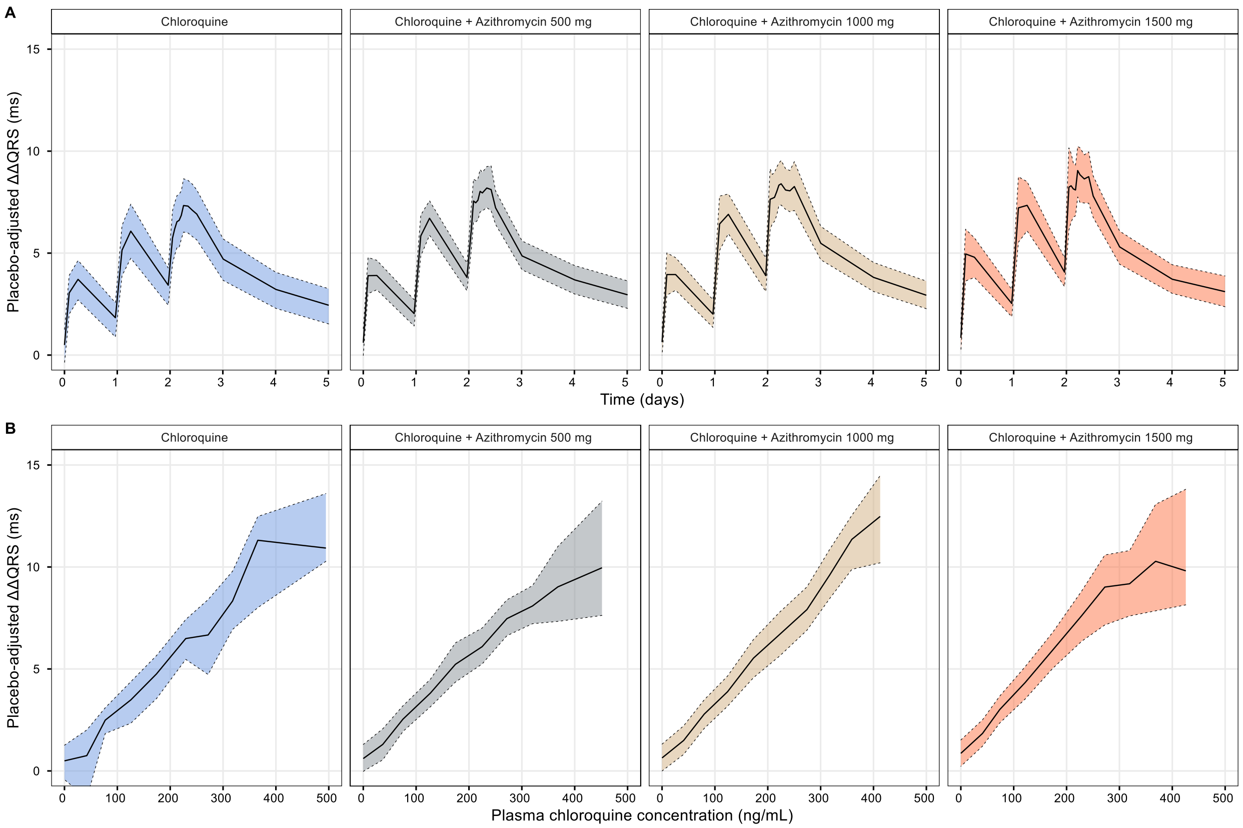

Figure S18: Placebo-adjusted ΔΔQRS interval, stratified by treatment arms; (A) ΔΔQRS interval vs. time after the first dose and (B) ΔΔQRS interval vs. observed plasma chloroquine concentrations. Solid lines represent the predicted median ΔΔQRS interval. Shaded areas represent the predicted 90% confidence interval.

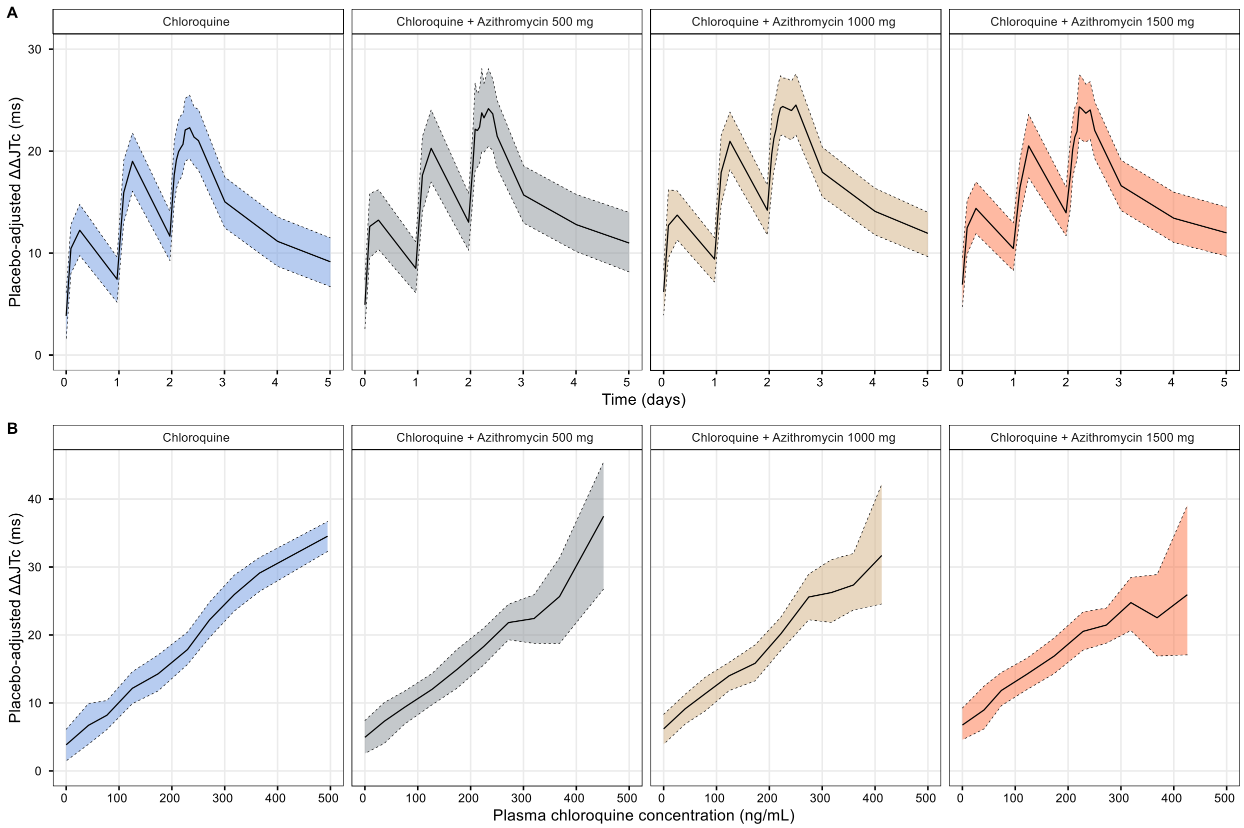

Figure S19: Placebo-adjusted ΔΔJTc interval, stratified by treatment arms; (A) ΔΔJTc interval vs. time after the first dose and (B) ΔΔJTc interval vs. observed plasma chloroquine concentrations. Solid lines represent the predicted median ΔΔJTc interval. Shaded areas represent the predicted 90% confidence interval.

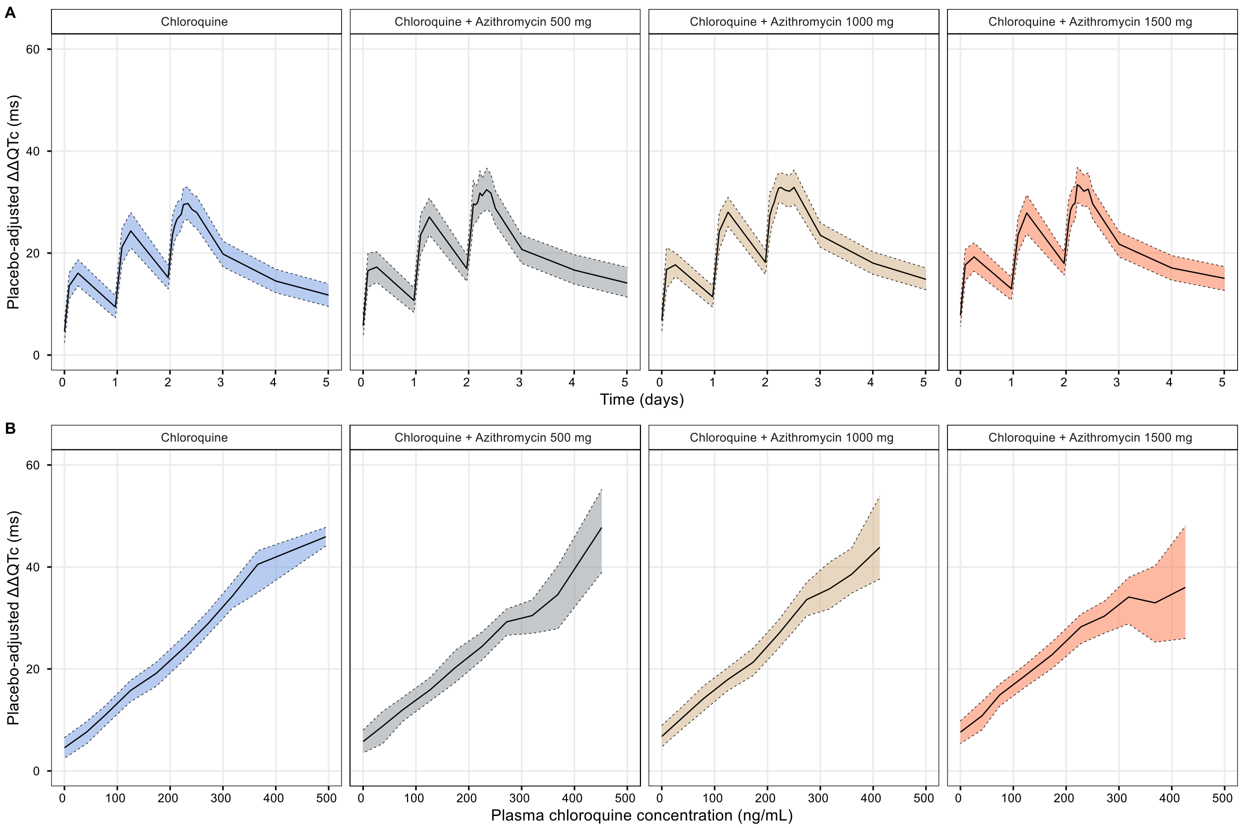

Figure S20: Placebo-adjusted ΔΔQTc interval, stratified by treatment arms; (A) ΔΔQTc interval vs. time after the first dose and (B) ΔΔQTc interval vs. observed plasma chloroquine concentrations. Solid lines represent the predicted median ΔΔQTc interval. Shaded areas represent the predicted 90% confidence interval.

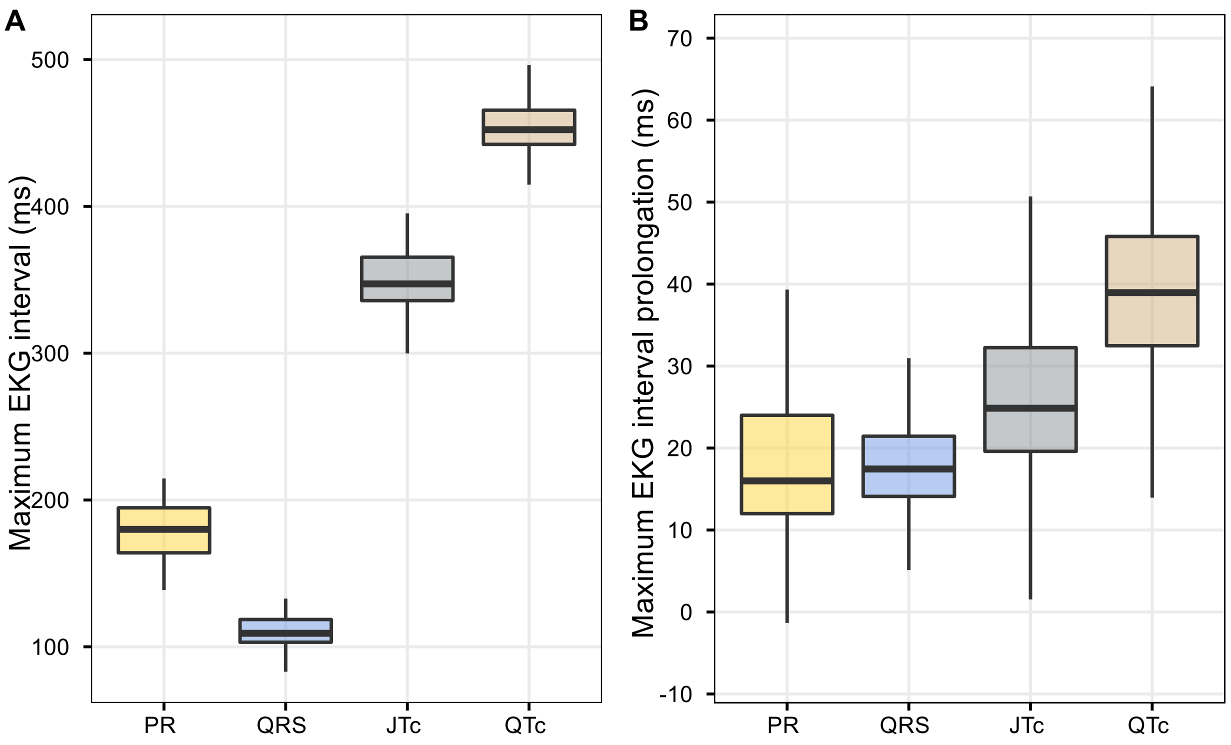

Figure S21: Box plot of the maximum electrocardiographic interval (A) and the maximum electrocardiographic interval prolongation (B), combining all arms of chloroquine phosphate (600 mg base) with or without azithromycin (n = 95 healthy adult volunteers). The QRS, JT, and QT intervals have been individually heart rate-corrected. The mean (SD; range) of the maximum changes on PR, QRS, JTc, and QTc are 18.4 (8.80; -1.33 to 39.33) ms, 12.1 (3.55; 3.66 to 21.3) ms, 30.9 (10.8; -2.02 to 61.8) ms, and 38.8 (11.4; 6.66 to 69.11) ms, respectively.

Table S1 Mean (SD) baseline participant characteristics, stratified by treatment arm.

	**Overall (n=119)**	**Placebo (n=24)**	**CQ alone (n=25)**	**CQ + 500 mg AZT (n=23)**	**CQ + 1000 mg AZT (n=24)**	**CQ + 1500 mg AZT (n=23)**
Age (years)	35.0 (13.2)	31.8 (14.1)	34.6 (13.4)	34.7 (12.2)	40.2 (12.3)	33.4 (13.2)
Body weight (kg)	83.2 (14.5)	83.3 (14.9)	88.1 (15.5)	82.9 (12.2)	83.9 (15.1)	77.6 (13.8)
Sex, male (n)	98	21	22	18	18	19
Electrocardiographic measurements (pooled all measurements at the day -1)						
RR interval (ms)	1011 (164)	1011 (135)	1028 (154)	1031 (194)	979 (169)	1007 (159)
PR interval (ms)	162 (21)	166 (27.6)	157 (18.4)	169 (19.2)	161 (17.7)	161 (22.9)
QRS interval (ms)	92.1 (9.89)	91.6 (9.27)	93.9 (9.72)	92.1 (1051)	91.4 (8.56)	91.9 (11.1)
JTc interval (ms)	324 (19.4)	322 (19.9)	321 (18.0)	326 (19.9)	326 (19.9)	328 (18.4)
QTc interval (ms)	417 (16)	413 (15.6)	415 (14.9)	419 (17.9)	417 (16.8)	420 (14.2)

CQ: chloroquine, AZT: azithromycin.
